# Stiff matrix induced srGAP2 tension gradients control migration direction in triple-negative breast cancer

**DOI:** 10.7150/thno.77313

**Published:** 2023-01-01

**Authors:** Chen Li, Zihui Zheng, Xiang Wu, Qiu Xie, Ping Liu, Yunfeng Hu, Mei Chen, Liming Liu, Wangxing Zhao, Linlin Chen, Jun Guo, Ying Song

**Affiliations:** 1 School of Medicine & Holistic Integrative Medicine, Nanjing University of Chinese Medicine, Nanjing 210023, Jiangsu, PR China.; 2 Key Laboratory of Drug Target and Drug for Degenerative Disease, Nanjing University of Chinese Medicine, Nanjing 210023, Jiangsu, PR China.; 3 Department of Anesthesiology, The Affiliated Hospital of Medical School, Ningbo University, Ningbo, 315040, PR China.; 4 Department of Respiratory and Critical Care Medicine, The Affiliated Changzhou No.2 People's Hospital of Nanjing Medical University, Changzhou 213003, People's Republic of China.; 5 Department of Pathology, Xuzhou Central Hospital, Xuzhou 221009, PR China.; 6 Department of Respiratory Medicine, The First Affiliated Hospital of Nanjing Medical University, Nanjing, China.

**Keywords:** srGAP2, polarized tension, stiff-directed migration, FRET, syndecan-4

## Abstract

**Rationale:** Cells migrating through interstitial matrix enables stiffening of the tumor micro-environment. To overcome the stiff resistance of extracellular matrix, aggressive cells require the extracellular mechanosensory activation and intracellular tension response. Mechanotransduction linker srGAP2 can synergistically control the mechanical-biochemical process of malignant cell migration.

**Methods:** To mimic the tumor micro-environment containing abundant collagen fibers and moving durotaxis of triple-negative breast cancer cells, the stiff-directed matrix was established. The newly designed srGAP2 tension probe was used to real-time supervise srGAP2 tension in living cells. The phosphorylation sites responsible for srGAP2 tension were identified by phosphorylated mutagenesis. Transwell assays and Xenograft mouse model were performed to evaluate TNBC cells invasiveness *in vitro* and *in vivo*. Fluorescence staining and membrane protein isolation were used to detect protein localization.

**Results:** The present study shows srGAP2 serves as a linker to transmit the mechanical signals among cytoskeleton and membrane. SrGAP2 exhibits tension gradients among different parts in the stiff-directionally migrating triple-negative breast cancer cells. Cells showing the polarized tension that increased in the leading edge move faster, particularly guided by the stiff interstitial matrix. The srGAP2 tension-directed cell migration results from the upstream events of PKCα-mediated phosphorylation at Ser^206^ in the F-bar domain of srGAP2. In addition, Syndecan-4 (SDC4), a transmembrane mechanoreceptor protein, drives PKCα regional recruit on the area of membrane trending deformation, which requires the distinct extent of extracellular mechanics.

**Conclusion:** SDC4-PKCα polarized distribution leads to the intracellular tension gradient of srGAP2, presenting the extra- and intracellular physiochemical integration and essential for persistent cell migration in stiff matrix and caner progression. Targeting the srGAP2-related physicochemical signaling could be developed into the therapeutic strategies of inhibiting breast cancer cell invasion and durotaxis.

## Introduction

Increased micro-environmental stiffness is closely associated with enhanced invasiveness, driving breast cancer 1, 2. The presence of thick aligned collagen fibers at the invasive front of primary tumor contributes to the worse cancer progression 3. The highly stiff collagen structure is a “highway” for cancer cells to invade and metastasize, due to the reduced cell membrane stiffness and protrusion formation at the cell front 4, 5. These membrane deformation-dependent phenotypes are highly controlled by mechanical forces of transmitting cytoskeleton tension to the membrane 6-8.

Bar-domian proteins (BDPs) serve as mechanotransduction linker, which can physically connect cytoskeleton and cell membrane, and determine the extent of membrane bending 9. These are essential for many actin-based cellular behaviors, such as podosome assembly and filopodium formation 10, 11. Slit-Roundabout (Robo) GTPase-activating protein 2 (srGAP2) is a family member of BDPs, involved in the outward bending of cell membrane 12. Lines of evidence demonstrate alternation of srGAP2 expression pattern closely associated with the cancer progression. High srGAP2 expression facilitates hepatocellular carcinoma cell migration 13. SrGAP2 contains conserved domains: N-terminal F-BAR, central Rho-GAP and a C-terminal SH3 domain. The F-BAR domain is membrane-binding modules through electrostatic-charge interaction between their positively charged amino acids (arginine and lysine) and the negatively charged phospholipids of the membrane, such as PtdIns (4,5) P2 and phosphatidylserine (PtdSer). The SH3 domain of srGAP2 can be combined with nucleation promoting factors (such as FMNL1/3), further bound on microfilament (MF) 14, 15.

During the stiff matrix-directed cell migration, transmembrane tension-sensing proteins are responsible for the delivery of extracellular mechanical cues into cells 16. Syndecan-4 (SDC4), a transmembrane adhesive protein, with the extracellular structure linked to extracellular matrix (ECM) and the intracellular structure linked to Talin 1 forms a protein complex attached to microfilaments (MFs) 17. SDC4 on the cell membrane specifically recruits and activates PKCα, contributing to mechanosensory activation and tension response in cells 18. Studies have suggested that the BAR domain is the mechanical sensitive region of BDPs, and phosphorylation of BAR domain could determine BDP biological function 19, 20. However, whether BAR domain phosphorylation participated in mechanical transmission and functional regulation of BDPs remains to be clarified.

The present study suggested that srGAP2 acts as a mechanotransduction protein across cell membrane and cytoskeleton, involved in malignant cell migration and cancer process. We have found srGAP2 tension gradients are necessary to maintain the direction and speed of cell movement. Membrane adhesion receptor SDC4 recruits PKCα, transferring the extracellular mechanical cues into cells, further induces the tension signal response by phosphorylating srGAP2. We propose that cell directional migration in the stiff micro-environment is controlled by the meshwork of chemical-mechanical signals, composing of membrane adhesion receptor, kinase and cytoskeleton-linker protein srGAP2.

## Materials and methods

### Cell cultures

293T, NIH 3T3, MDA-MB-231 and MCF7 cell lines were acquired from the American Type Culture Collective (Manassas, Virginia, USA). Short tandem repeat (Genetic Testing Biotechnology Corporation, Nanjing, China) analysis was performed for gene identification. MDA-MB-231 cells were cultured in Leibovitz L-15 Medium (Gibco), containing 10% fetal bovine serum (Gibco) and antibiotics (100 U/mL streptomycin penixillin). 293T, NIH 3T3 and MCF7 cells were cultured in Dulbecco's Modified Eagle's Medium (Gibco, Grand Island, NY, USA) containing 10% FBS (Gibco) and antibiotics (100 U/mL streptomycin penixillin). All the cells were grown under humidified air containing 5% CO₂ at 37 °C.

### Biochemical agents and antibodies

Recombinant human epidermal growth factor (EGF) and C-X-C motif chemokine 12 (CXCL12) was acquired from Sigma-Aldrich (E5036 and SRP4391). Cytochalasin D (Cyto D) was obtained from MilliporeSigma (Burlington, MA, USA). Blebbistatin (Bleb) was from Aladdin (Shanghai, China). PMA (PKC activator; HY-18739), Go 6983 (PKC inhibitor; HY-13689) and LY-294002 (PI3K inhibitor; HY-10108) were obtained from MedChemExpress. The following antibodies were used: Rabbit anti-srGAP2 (Proteintech, #22519-1-AP); Rabbit anti-β-actin (CST, #4970); Mouse anti-actin (Abcam, #ab179467); Mouse anti-p-Ser (Santa, #sc-81514); Rabbit anti-PKCα (Abcam, #ab32376); Rabbit anti-syndecan-4 (Abcam, #ab74139); Mouse anti-yndecan-4 (Santa, #sc-12766); Rabbit anti-FMNL1 (Proteintech, #27834-1-AP); Rabbit anti-ATP1A1 (Proteintech, #14418-1-AP); Rabbit anti-FMNL3 (Sigma, #PA5-46742) and Mouse anti-Talin 1 (Abcam, #ab108480).

### Plasmid generation and transfection

The pCMV-srGAP2-GFP (CG90484-ACG), pCMV-PKCα-OFP (HG10026-ANR), pCMV-PKCα-Flag (HG10026-NF) and pCMV-syndecan-4-GFP (MG50726-ACG) were acquired from SinoBiological (Beijing, China). In accordance with previous reports 21-23, the angle-dependent FRET-based tension probe was created using circularly permutated cpVenus and cpCerulean (cpstFRET) [mTurquoise2-7aa-super (s) YFP2 circularly permuted stretch-sensitive FRET (cpstFRET)], followed by its insertion between Pro485 and Pro486 of srGAP2. The mutation plasmids (srGAP2-S80A, srGAP2-S110A, srGAP2-S206A, srGAP2-S220A and srGAP2-S206E) were constructed using KOD-Plus-Neo (TOYOBO, Osaka, Japan). All the Plasmids were then extracted from individual colonies and purified with Endo-free Plasmid Mini Kit (OMEGA, USA). The integrity of all expressed structures was confirmed by DNA sequencing. Plasmids encoding srGAP2 tension sensor were transfected into MDA-MB-231 cells with Lipofectamine™ 3000 Transfection Reagent (Invitrogen, Carlsbad, CA, USA) and Opti-MEM™ media (Invitrogen, Carlsbad, CA, USA) according to manufacturer instructions. The transfection efficiency was about 70%.

### The small interfering RNA (siRNA) design

The small interfering RNA targeting srGAP2, FMNL1, FMNL3, PKCα, SDC4 and the negative control siRNA were designed and synthesized by Genepharma (Shanghai, China).

- srGAP2 sense: 5′-CCACUCAUCCCUGAAGAAUTT-3′, Anti-sense: 5′-AUUCUUCAGGGAUGAGUGGTT-3′.
- FMNL1 sense: 5′-CCUGGUGAAGGUCAUUGCU-3′, Anti-sense: 5′-AGCAAUGACCUUCACCAGG-3′.
- FMNL3 sense: 5′-CUGUCAGCCAUUCGAAUU-3′, Anti-sense: 5′-UAAUUCGAAUGGCUGACAG-3′.
- PKCα sense: 5′-CAACGUACCCAUUCCGGAAtt-3′, Anti-sense: 5′-UUCCGGAAUGGGUACGUUGta-3′.
- syndecan-4 sense: 5′-GGCACCUUAAUGCUGACUUTT-3′, Anti-sense: 5′-AAGUCAGCAUUAAGGUGCCTG-3′.

### Acquisition of clinical samples and bioinformatic analyses

Fresh TNBC tissue and adjacent normal tissue samples were surgically obtained from the Department of Pathology, Central Hospital of Xuzhou (China). Informed consent was obtained from all patients, and ethical approvals were obtained from Ethics Committee of Xuzhou Central Hospital. Survival analysis was performed through the Kaplan-Meier plotter database (http://kmplot.com/analysis/), and log-rank test was used for significance testing. Modules exploring associations between *SRGAP2* gene expression and tumor features in TCGA was performed through the Timer2.0 database (http://timer.comp-genomics.org/).

### FRET image acquisition and analysis

FRET image acquisition was performed using a confocal microscope (SP8; Leica, Wetzlar, Germany) equipped with a ×63 oil-immersion objective lens. Emission spectra were sorted using dual-view-2 (DV2, MAG Biosystems), and cyan and yellow emission wavelengths (EM) were detected [excitation wavelength (EX) = 436 nm and EM = 535/30 nm for CFP detection and 470/30 nm for YFP detection]. Cells were imaged under the constant exposure (400 ms) and gain (2.0). The dipole angle between the donor/CFP and the acceptor/YFP determined FRET effectiveness.

FRET images were analyzed using Fiji software 24. For the signal cell FRET analysis, FRET/Acceptor emission ratios were calculated for each pixel clearest optical plane for each image field. The cell was first selected by generating a binary mask using the drawing tool in Fiji. A Donor mask was generated by applying a threshold on the Donor image, which was then made binary by converting pixel intensity values greater than the Donor threshold to 1 and those lower than it to 0. A similar mask was generated for the acceptor channel. Ratio images (32-bit) were calculated the CFP/FRET ratio (the intensity of the CFP channel divided by the intensity of the FRET channel) using the equation E = eCFP donor/eYFP acceptor, which correlated negatively correlated with FRET efficiency, but positively with force. For presentation purposes, pseudocolour was applied using Fiji software in order to obtain the final images.

### Fluorescence Recovery after Photobleaching (FRAP) test and acceptor-bleaching FRET (FRET-AB) test

In the FRAP test, we selected a region of interest (ROI) and bleached it with a 590 nm laser at 100%. Time-series images were acquired before and after bleaching in 500 s, then we recorded the fluorescence intensity in the ROI and calculated the fluorescence recovery rates. The fluorescence intensities obtained were normalized to the average pre-bleach values in the GraphPad Prism software.

In a FRET-AB test, we bleached the acceptor with a 514 nm laser at 100% 20 times, and then the fluorescence intensity of the donor and acceptor after bleaching was recorded. We calculated the FRET efficiency using the equation E= (Edonor-after- Edonor-before)/Edonor-before. Cells were imaged using the FRAP or FRET-SE model of Lecia SP8 confocal microscopy with a 63× objective lens under the constant exposure (400 ms) and gain (2.0) 21, 25.

### Stiff-directed matrix production and chemotaxis assay

According to the method developed by Yamada Laboratory 26, 27, cell-derived matrices (CDM) were generated. In brief, a 35 mm confocal dish (Corning) was pre-washed with 150 ml 50 mg/ml polylysine PDL (Sigma Aldrich) for 20 min. After that, the bottom of the confocal dish was coated with 0.2% gelatin (Sigma Aldrich, #V900863) and crosslinked with 0.1% glutaraldehyde, quenched with 1 M glycine (Biofroxx, #1275GR500). Then NIH 3T3 cells (fibroblasts, 2 × 10^4^ cells/cm^2^) were seeded and grown for 8 days in DMEM containing 10% FBS and 50 μg/ml ascorbic acid (Sigma Aldrich, #A92902). Matrices were denuded of living cells by incubation with PBS containing 20 mM NH₄OH and 0.5% Triton X-100, and DNA residue was removed by incubation with DNaseI.

CDM with stiffness differential are created by softening a specific area of the CDM using magnetic beads (Takara) coated with trypsin, as trypsin has been demonstrated to modify CDM stiffness 28. Briefly, 10 ml of tryptin-coated magnetic beads are added to the CDM with a pipette and secured with magnets to the corner between a fan-shaped area. The confocal dishes were incubated at 37 °C with 5% CO₂ for 10 min, and then rinsed with PBS 3 times. Within 24 h after the CDM was generated, about 2×10^4^ treated cells were planted at the starting site (the corner between the fan-shape softened by trypsin). After incubation at 37 °C for 1 h, live cell imaging was performed with confocal laser scanning microscopy (SP5; Leica, Wetzlar, Germany). The images were gained every 60 s. Five autonomous moving cells were randomly selected for each experiment, and their trajectory were recorded. The results were evaluated using Imaris 7.6.3 (Bitplane AG, Zurich, Switzerland).

### Cell migration and invasion assays

MDA-MB-231 cells were inoculated in a 96-well plate, and at 90% confluency, a special cell scraping device was used to gently scrape the monolayer of cells. The floating cells were removed by washing with PBS three times, and the remaining adherent cells with thin “wounds” were incubated at 5% CO₂ and 37 °C. A time-lapse imaging system was used to capture photographs every 1 h.

The transwell apparatus (Corning, USA) was pre-coated upper chamber with 30 μL of matrigel solution. MDA-MB-231 cells (2×10^5^) were starved for more than 12 h, inoculated in the upper compartment with serum-free media, with the presence of 20% fetal bovine serum in the lower compartment. After 24 h incubation, the cells that had infiltrated into the lower compartment were fixed and stained using 0.4% crystal violet. The stained cell images were used for cell invasion analysis.

### Immunofluorescence analysis

Cells were removed from their medium and washed three times with pre-cooled phosphate-buffered saline (PBS). Paraformaldehyde (4%) was used to immobilize cells for 20 min at room temperature. Remove paraformaldehyde and wash the cells with PBS for three times. The cells were treated with 0.1% Triton X-100 in PBS for 15 min at room temperature, blocked with 4% bovine serum albumin/PBS for 1 h at room temperature and incubated with primary antibodies overnight at 4 °C. Remove the primary antibody and wash the cells three times with pre-cold PBS. The cells were then incubated with secondary antibody at room temperature in dark for 1 h. DAPI was used to label the nucleus. The change in the fluorescence value was monitored under a Leica SP8 inverted fluorescence microscope (Leica, Wetzlar, Germany).

In the method of phalloidin staining after Triton X-100 permeabilization, cells were fixed first, incubated in PBS containing 0.3% Triton X-100 and 5% BSA for 1 h. After washed in PBS with 5% BSA, cells were stained with phalloidin in PBS with 5% BSA for 30 min.

### Western Blotting and immunoprecipitation

The cells were seeded in six-well plates and digested with a lysate containing phenylmethylsulfonyl fluoride (PMSF) (Roche, Basel, Switzerland) and a protease inhibitor cocktail (Millipore Sigma, St. Louis, MO, USA). The extracted total proteins were separated via SDS-PAGE and transferred to nitrocellulose membranes, which were incubated in 5% non-fat milk and blocked for 1 h. The membranes were then incubated with specific antibodies overnight at 4 °C. After washing three times, the membranes were incubated with secondary antibodies for 2 h. The enhanced chemiluminescence (ECL) chromogenic substrate was used to visualize the immunoreactive protein bands, and the protein band intensities were quantified using densitometry (Quantity One; Bio-Rad, Hercules, CA, USA). β-actin was set as the control. The cytosol and membrane protein fractions were isolated from the cells using Mem-PER plus membrane protein extraction kit (#89842, Thermo Fisher Scientific) as per manufacturer's protocol.

For co-IP experiments, cells were lysed in 0.5% NP-40 lysis buffer (50 mM Tris-HCl, pH 8.0, 150 mM NaCl, 0.5% NP-40, 1 mM DTT) with protease inhibitors cocktail (Selleck). After centrifugation at 16,200 g for 15 min, the supernatants were collected and incubated with primary antibody at 4 °C for 8 h followed by incubating with Protein-A beads (Millipore) for another 4 h at 4 °C. After incubation, samples were washed with lysis buffer for five times. 2 × loading buffer was added to the sample and heated at 95 °C for 5 min. After centrifugation, upper samples were collected.

### Stable cell lines and xenograft mouse model

MDA-MB-231 cells expressing GFP-tagged empty vector, GFP-srGAP2 WT and GFP-srGAP2 S206A were cultured in medium containing 1000 mg/mL geneticin to screen transfected cells for 7 days. If >90% cells were fluorescent, subsequent experiments were performed. Twenty 6-week-old female BALB/c nude mice (16-18 g) were obtained from the Institute of Comparative Medicine, Yangzhou University (China) and maintained under specific non-pathogenic conditions at Nanjing University of Chinese Medicine. The animals were randomly divided into three groups: empty vector, srGAP2 WT and srGAP2 S206A. Each group was injected *in situ* with MDA-MB-231 cells (1 × 10^6^) suspended in 20 µL matrigel. Lung nodules were monitored and quantified using ChemStudio PLUS (Jena, Jena, Germany) at different time points. On the 28th day, the mice were sacrificed and their organs were immediately removed to obtain evidence of metastatic signals. The lungs of these mice were stained with hematoxylin and eosin, and the lung nodules in serial sections were microscopically quantified.

The stable cell lines expressed GFP-vector or GFP-srGAP2 were then infected with knockdown lentivirus expressing sh-NC or sh-*SDC4* and selected with puromycin. After re-screening, we obtained three stable cell lines (GPF-vector + NC; GFP-srGAP2 + NC; GFP-srGAP2 + *SDC4* KD) for tumorigenesis in mice.

### Data statistics

Data are shown as the mean ± SEM. For comparisons of two groups, unpaired Student's t-test was used. For comparisons of three or more groups, one-way ANOVA test followed by Tukey's multiple comparisons post-test was used. All statistical analyses were performed using SPSS v.22.0 (IBM Corp. Armonk, NY, USA). The statistical analysis was described in each figure legend. Differences between or among groups were denoted as ns for not significant, * for *P* < 0.05, ** for *P* < 0.01, *** for *P* < 0.001.

## Results

### SrGAP2 cooperated with cytoskeleton tension to participate in matrix-directed cell migration

**Figure 1:**
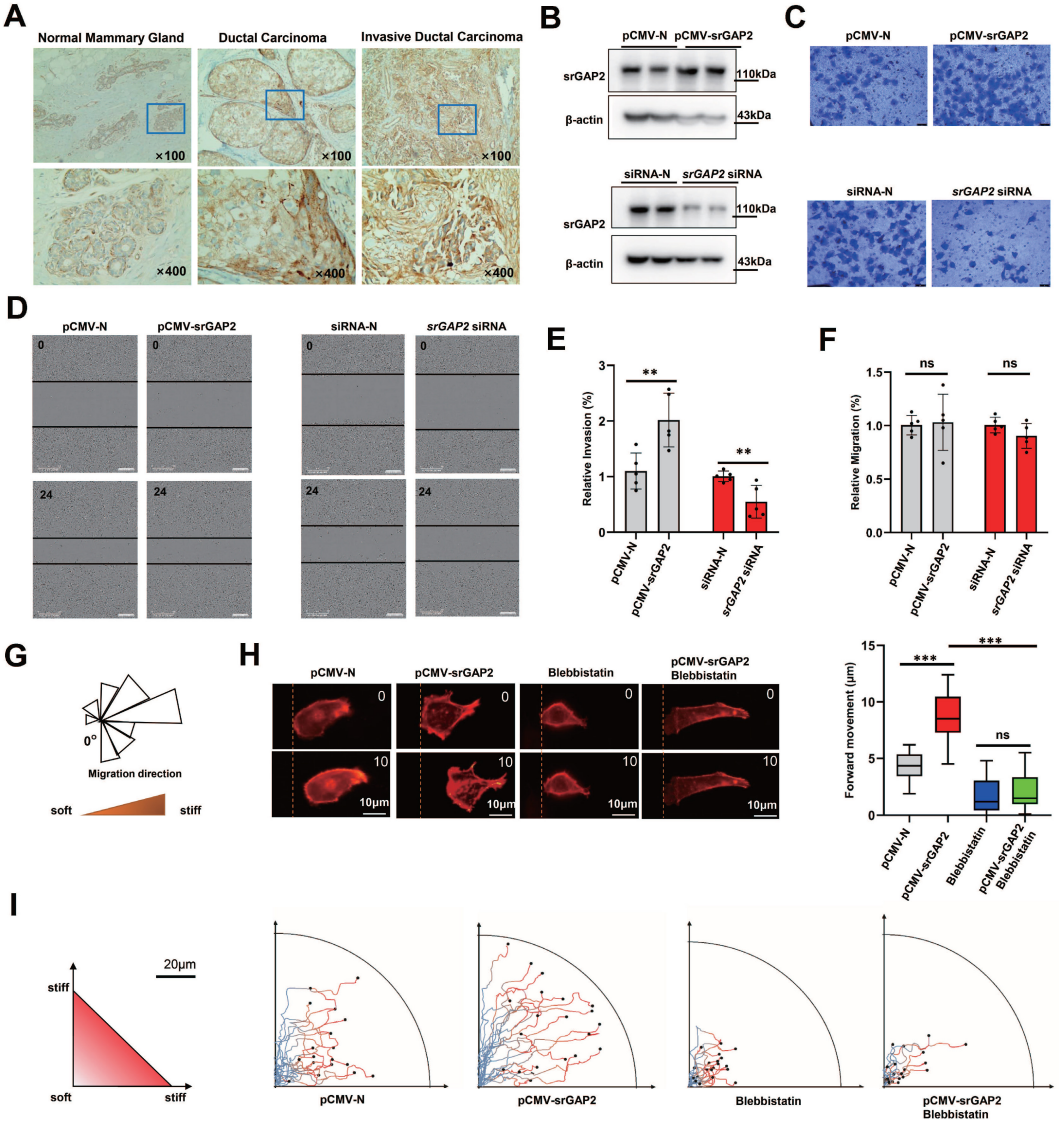
** SrGAP2 and MF tension synergistically promoted TNBC cell migration in stiff-directed matrix. (A)** Immunohistochemistry of srGAP2 in TNBC tissues with different histological grades (magnification, 100× and 400×). **(B)** Immunoblot of srGAP2 in MDA-MB-231 cells transfected with* pCMV-srGAP2* plasmid or *srGAP2*-siRNA. **(C)** Invasion of MDA-MB-231 cells determined by transwell invasion assay. **(D)** Migration of MDA-MB-231 cells determined by scratch wound assay. **(E)** Quantification of cell invasion corresponding to the Figure 1C (mean ± SD, n = 5 experiments. Statistical analysis was performed using the unpaired Student's t-test, **P <​ 0.01). **(F)** Quantification of cell migration from the scratch wound results, corresponding to the Figure 1D (mean ± SD, n = 5 experiments. Statistical analysis was performed using the unpaired Student's t-test, ns: no statistical significance). **(G)** MDA-MB-231 cells in stiff-directed matrix, rose plot shows the direction of cell migration (locally softened matrix at "0") (n > 12 cells, 5 repeats). **(H)** Confocal sections of MDA-MB-231 cells in stiff-directed matrix were shown 600 s apart, the dashed yellow line indicated the start position (scale bar: 10 μm). The distance of forward movement (μm). One-way analysis of variance was used for the single-factor sample comparisons. ***P <​ 0.001.** (I)** Live cell imaging shows the migration tracks of randomly-selected cells in stiff-directed matrix within 1 h (n = 20 cells, 3 repeats).

Clinical data (TCGA database) shows increased *SRGAP2* expression associated with high-grade and aggressive tumors (Figure S1A). Triple-negative breast cancer patients with high *SRGAP2* expression suffer poor prognosis (Figure S1B). Immunochemistry of ductal carcinoma of breast showed that srGAP2 aggregation was more pronounced in “budding” cancer cells, which was invading into the surrounding basement membrane; srGAP2 expression was significantly increased in the invasive ductal carcinoma (Figure 1A). We next investigated whether srGAP2 directly regulates Triple-Negative Breast Cancer (TNBC) cells invasion *in vitro*. In TNBC cells, srGAP2 was overexpressed by transfection of srGAP2 plasmid, or SRGAP2 was silenced by small interfering RNA (Figure 1B). Transwell assay showed a positive correlation between srGAP2 expression and the invasiveness of TNBC cells (Figure 1C and 1E). Taken together, these results suggested that srGAP2 was correlated closely with the malignant behavior of TNBC.

Cell scratch assay *in vitro* presented srGAP2 expression not affect 2D migration of TNBC cells (Figure 1D and 1F). However, to mimic the tumor micro-environment containing abundant collagen fibers and the tendency of tumor cells to the stiff matrix, we used magnetic trypsin-coated beads to soften the local matrix without damaging its structure, so as to generate stiff-directed matrix (Figure 1G, 29, 30). A Real-time fluorescence imaging system was used to record cell trajectories. When cells move towards the stiffer matrix, srGAP2 overexpression drived cells move faster (Figure 1H) and continuous route (Figure 1I). In addition, myosin II inhibitor blebbistatin-induced elimination of MF tension showed disorganized and blocked migration, completely antagonized srGAP2-induced directed motion (Figure 1H-I). These suggested that srGAP2 promotes fast-moving cells in stiff-directed matrix depending on MF tension.

### The physical junctions mediated by srGAP2 transmitted mechanical forces between MF and cell membranes

**Figure 2:**
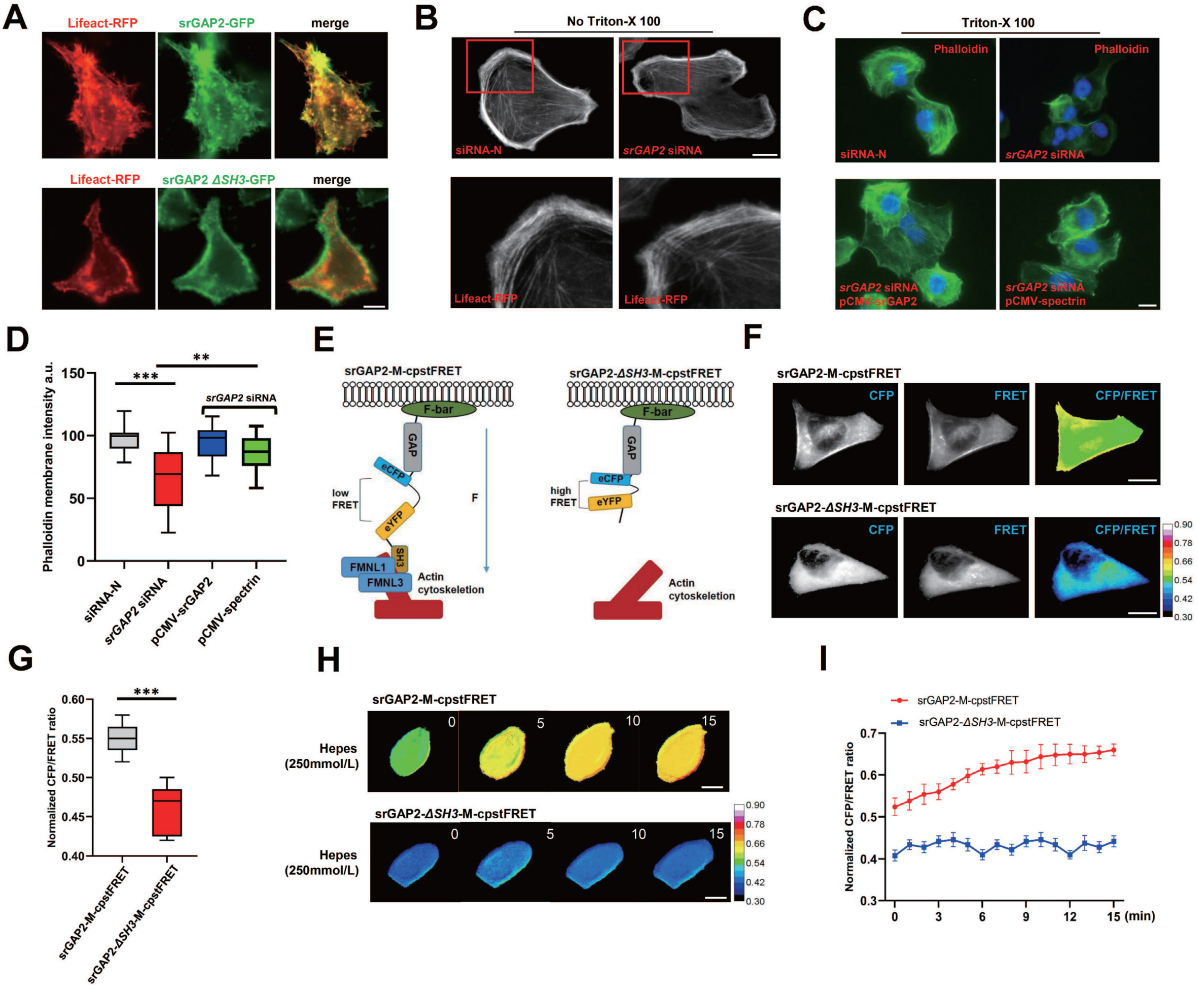
** SrGAP2 was required for attaching MF to cell membrane. (A)** MDA-MB-231 cells were transfected with plasmids expressing the GFP-labeled *srGAP2* (or *SH3* domain deletion, *srGAP2 ΔSH3*) and* Lifeact. Sc*ale bar: 10 μm. **(B)** MDA-MB-231 cells were transfected with non-targeting siRNA (siRNA-N) or *srGAP2* siRNA, and MF were labeled with lifeact-RFP. Images were taken by confocal microscopy. Scale bar: 10 μm. **(C)** Triton X-100 permeabilized MDA-MB-231 cells transfected with siRNA-N or *srGAP2* siRNA, and supplementary expression of *srGAP2* or* spectrin* plasmid. Phalloidin was used to label cortical actin. Scale bar: 10 μm. **(D)** Quantification of phalloidin membrane intensity. One-way analysis of variance was used for single-factor sample comparisons. **P <​ 0.01 and ***P <​ 0.001.** (E)** The angle-dependent FRET-based tension probe using circularly permuted cpVenus and cpCerulean (cpstFRET), followed by its insertion between srGAP2 GAP domain and SH3 domain. No force control was constructed by deletion of the SH3 domain (actin-binding domain) to block force transmission between actin and srGAP2 tension sensor. **(F** and **G)** FRET representative images of MDA-MB-231 cells harboring srGAP2-M-cpstFRET and srGAP2 *ΔSH3*-M-cpstFRET probes. Scale bar: 10 μm. The means of CFP/FRET ratios are shown (mean ± SD, n = 20. Statistical analysis was performed using the unpaired Student's t-test, ***P <​ 0.001). **(H)** Time-lapsing imaging of srGAP2 tension and srGAP2 *ΔSH3* tension in cells treated with hypotonic buffer (Hepes: 250 mmol/L). Scale bar: 10 μm. **(I)** Normalized signals corresponding to srGAP2 tension and srGAP2 *ΔSH3* tension versus time, respectively (mean ± SEM, n ≥ 5). The calibration bar: 0.3 to 0.9 for srGAP2-M-cpstFRET and srGAP2 *ΔSH3*-M-cpstFRET probes.

To further investigate the mechanism underlying srGAP2 promoting tumor cell migration in stiff-directed matrix, GFP-labeled *SRGAP2* was transfected into MDA-MB-231 cells. We found strong co-localization of GFP-srGAP2 and Lifeact-tagged MF, but co-localization disappeared when srGAP2 failed to bind with MF (Figure 2A, *SH3* domain deletion). We considered that cytoskeleton-dependent mechanical changes might be responsible for srGAP2 involved in tumor cell migration.

Firstly, we found that actin polymerization appeared not change in the *SRGAP2* knockdown group (Figure 2B). Furthermore, using actin tension probes, we found that the loss of srGAP2 did not change the MF tension (Figure S2A). Next, we studied whether srGAP2 acts as physical-connecting structures between MF and cell membranes. The loss of srGAP2 compromises the transmission of actomyosin contraction forces to cell membranes and further attenuates cell motility. To investigate this hypothesis, in Triton X-100 permeabilized cells, we visualized F-actin by phalloidin staining and found a dramatic decrease in cortical actin levels in the SRGAP2 knockdown group. The results suggested that the strong interaction between cytoskeleton and plasma membrane, normally resistant to solubilization by Triton X-100, was compromised 9, 31. The level of cortical actin in cells was restored after supplementary expression of srGAP2 WT plasmid. In addition, we found that the additional complement of spectrin, another cytoskeleton-membrane linker protein 32, also restored membrane-cytoskeleton junctions (Figure 2C-D). These results further support the key role of srGAP2 in transmitting force between MF and cell membrane.

### Construction and test of the srGAP2 tension probe

To detect real-time changes in srGAP2 tension, we designed a FRET-based tension probe. The FRET module was incorporated within the srGAP2 backbone, which reports the real-time resonant energy transfer during angle twisting of the donor-acceptor pair induced by tension loading onto srGAP2 (Figure S3A-B). FRET-AB test was used to verify the FRET efficiency of srGAP2 tension probe. The acceptor fluorescence (eYFP) was decreased dramatically upon acceptor photobleaching (AB). Meanwhile, the donor fluorescence (eCFP) was increased due to the unacceptable energy transfer from donor to acceptor after photobleaching (FRET AB = 28.46%, Figure S3C). This experiment showed the efficiency of FRET events between the two fluorophores. In the fluorescence recovery after photobleaching (FRAP) test, the recovery rates for the srGAP2 in region of interest (ROI) was 49.10% at 500 s after bleaching (Figure S3D-E). To distinguish force-dependent from force-independent FRET changes, we also constructed srGAP2 probes knocking out SH3 domain as the srGAP2 no force control, whose failed to bind with MF (Figure 2E). FRET analysis showed that in MDA-MB-231 cells, the tension load of srGAP2-M-cpstFRET was significantly higher than that of srGAP2-*ΔSH3*-M-cpstFRET (Figure 2F-G). To analyze the sensitivity of srGAP2-M-cpstFRET in tension load, we altered cytoskeletal tension with a hypotonic buffer. Time-lapse imaging was performed for 15 min, which presented a gradual increase in tension loading on srGAP2-M-cpstFRET. Importantly, no changes in FRET ratio were observed for the respective srGAP2 no force control (Figure 2H-I). Based on the above results, the srGAP2-M-cpstFRET could be used to detect real-time srGAP2 tension during cell movement.

**Figure 3:**
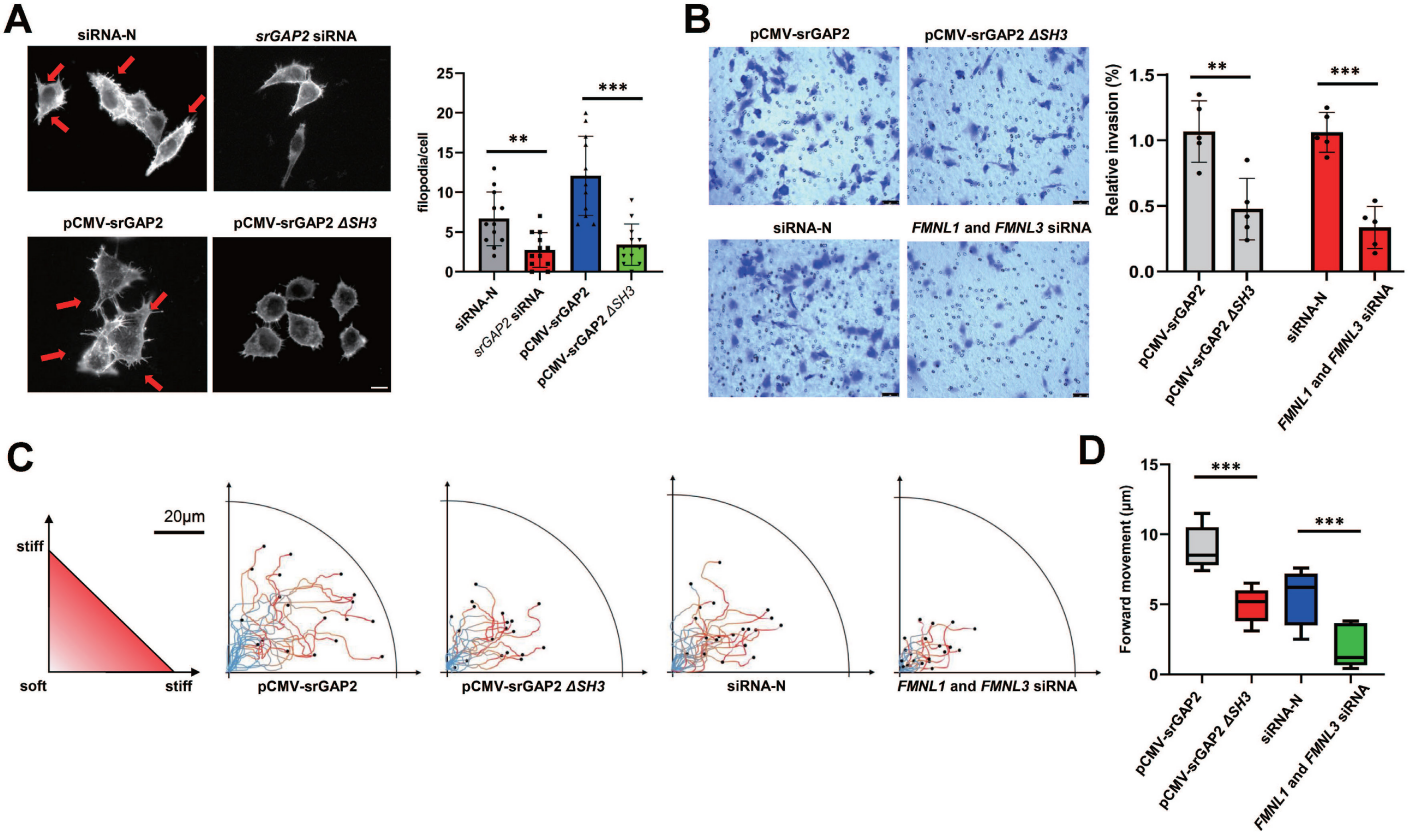
** SrGAP2 tension transmitting from MF was responsible for cell invasion and persistent migration in stiff-directed matrix. (A)** Representative images of MF structures in cells transfected with non-targeting siRNA or *srGAP2* siRNA, harboring srGAP2-WT or srGAP2 *ΔSH3*. FITC-stained MFs; nucleus: blue; white arrows: filopodia and lamellipodia structures. Scale bar: 10 μm. The number of filopodia per cell was quantified (length >​ 1 μm, n >​ 20 cells. Statistical analysis was performed using the unpaired Student's t-test, **P <​ 0.01 and ***P <​ 0.001). **(B)** Invasion of MDA-MB-231 cells harboring srGAP2 WT and srGAP2 *ΔSH3*, or transfected with non-targeting siRNA, and *FMNL1*-*FMNL3* siRNA determined by the transwell invasion assay. Quantification of cell invasion (mean ± SD, n = 5 experiments. Statistical analysis was performed using the unpaired Student's t-test, **P <​ 0.01 and ***P <​ 0.001). **(C)** Live cell imaging showed the migration tracks of cells harboring the distinct plasmid or siRNA in stiff-directed matrix within 1 h (n = 20 cells, 3 repeats). **(D)** The distance of forward moving MDA-MB-231 cells in stiff-directed matrix within 10 min. One-way analysis of variance was used for single-factor sample comparisons. ***P <​ 0.001.

To further investigate the role of srGAP2 tension in membrane deformation and cell migration, the knockdown of srGAP2 ligand proteins FMNL1 and FMNL3 were transfected to weaken srGAP2 and F-actin binding (Figure S2B, 15, 33). We found that srGAP2 no force control reduced the number and length of filopodia (Figure 3A). Transwell assay showed that the loss of srGAP2 tension decreased the TNBC cells invasion (Figure 3B). In the stiff-directed matrix, analysis of cell movement trajectory showed that srGAP2-MF disconnection significantly reduced cell motility (Figure 3C-D). These further confirmed that srGAP2 tension rather than cellular localization was particularly involved in matrix-directed cell migration.

### Higher srGAP2 tension correlated with pseudopodia formation, and the polarized srGAP2 tension regulated the matrix-directed cell migration

**Figure 4:**
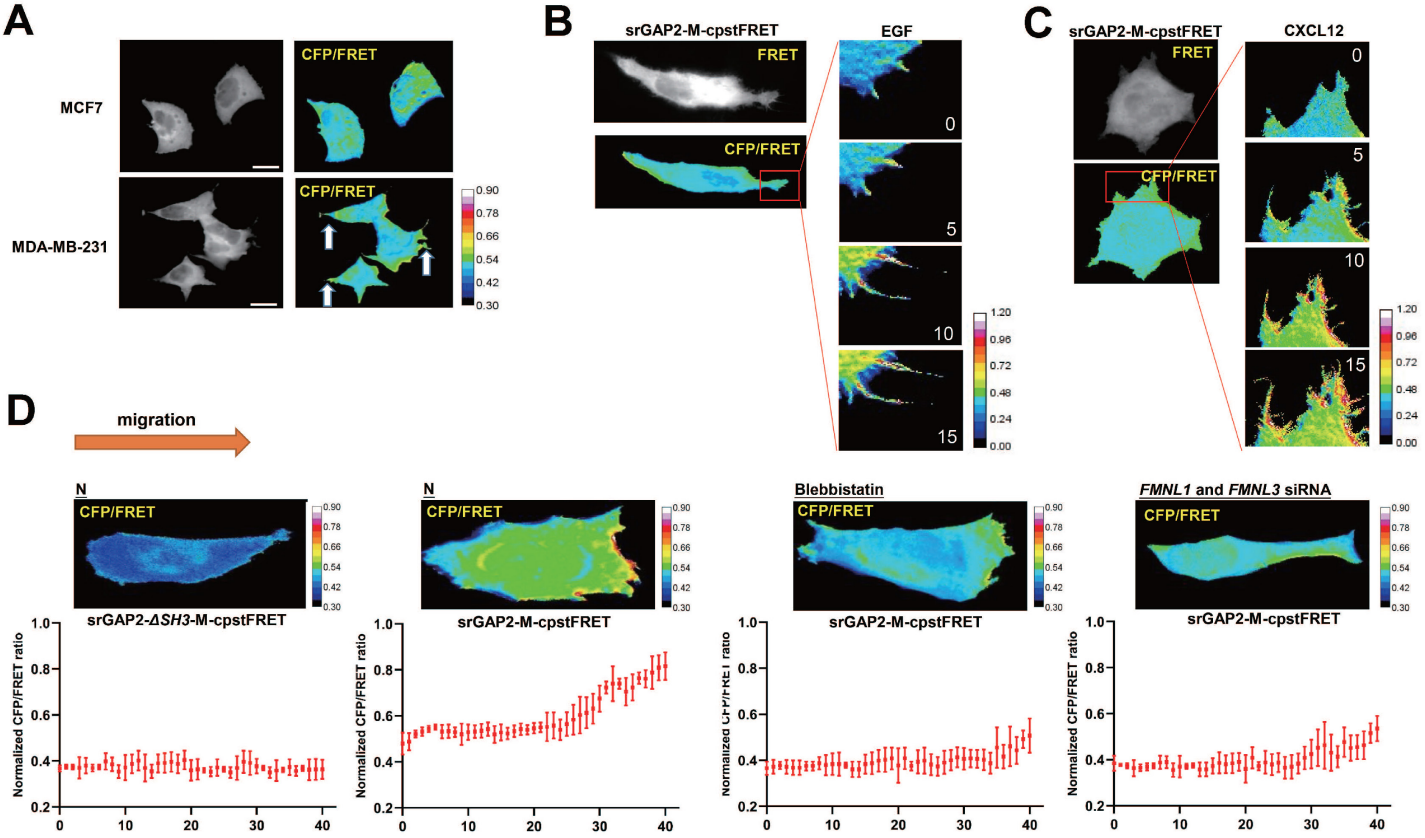
** SrGAP2 tension in fast-moving cells. (A)** FRET images of MCF7 and MDA-MB-231 cells expressing srGAP2-M-cpstFRET probes (white arrows: filopodia and lamellipodia structures). The calibration bar: 0.3 to 0.9.** (B)** and **(C)** FRET time-lapse images of MDA-MB-231 cells expressing the *srGAP2-M-cpstFRET* probe after treatment with EGF (10 ng/ml) or CXCL12 (500 ng/ml) for 15 min. The calibration bar: 0.0 to 1.2. **(D)** MDA-MB-231 cells expressing *srGAP2 ΔSH3-M-cpstFRET* and *srGAP2-M-cpstFRET* probes in stiff-directed matrix (the orange arrow at the top: the direction of cell migration). Cells were equally divided into forty parts along with the moving direction, and the CFP/FERT of each part was statistically quantified to present the srGAP2 tension in this part. The calibration bar: 0.3 to 0.9.

Since pseudopodia formation determines cell migration and invasion, we further investigated srGAP2 tension in human breast cancer cells MCF7 (barely invasive) and MDA-MB-231 cells (highly invasive). SrGAP2 tension was uniformly distributed in MCF7 cells, but in MDA-MB-231 cells, srGAP2 tension was significantly higher in the pseudopodia (Figure 4A). Furthermore, we treated MDA-MB-231 with EGF or CXCL12 for 15 min to induce filopodia generation and prolongation and the time-lapse photographs were captured in the MDA-MB-231 cells transfected with srGAP2-M-cpstFRET 34, 35. Interestingly, the srGAP2 tension increased significantly within filopodia extension, while its tension only slightly increased in the cell body (Figure 4B-C). Therefore, we propose that srGAP2 tension overcomes the high-energy barrier of membrane deformation and participates in the membrane bulges.

However, the tension of srGAP2 is quite different during cell moving in the stiff-directed matrix. FRET imaging of srGAP2 presented the tension gradient, with significantly higher srGAP2 tension in the cell front. When myosin II activity was inhibited (blebbistatin treatment) or the connection between srGAP2 and MF was broken (*FMNL1* and *3* siRNA), not only srGAP2 tension decreased in cells, but also the tension gradient disappeared (Figure 4D). These suggest the polarized srGAP2 tension necessary for fast movement of tumor cells in stiff-directed matrix.

### Phosphorylation dependent regulation of srGAP2 tension determines cell stiff-directed migration and pseudopod formation

**Figure 5:**
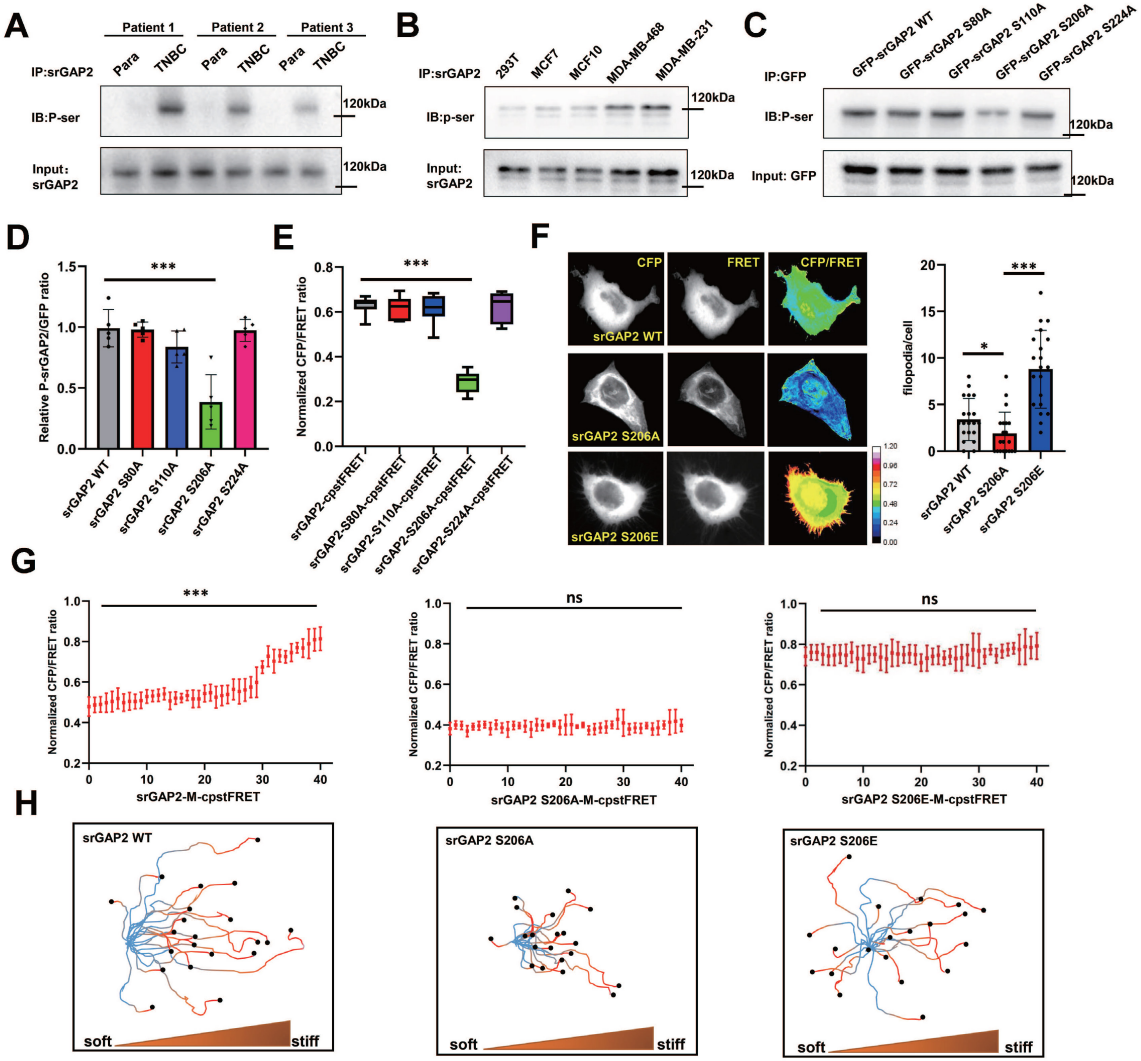
** Ser^206^ phosphorylation in the F-bar domain regulated srGAP2 tension. (A)** Endogenous srGAP2 was immunoprecipitated in the fresh human TNBC tissues and para-cancer tissues. Phosphoserine (p-Ser) and total srGAP2 were identified by western blot. **(B)** Endogenous srGAP2 was immunoprecipitated in 293T, MCF7, MCF10, MDA-MB-468 and MDA-MB-231 cell lines. Phosphoserine (p-Ser) and total srGAP2 were identified by western blot. **(C)** GFP-srGAP2 WT, GFP-srGAP2 S80A, GFP-srGAP2 S110A, GFP-srGAP2 S206A and GFP-srGAP2 S224A were expressed in MDA-MB-231 cells and the amount of phosphorylated srGAP2 was determined. **(D)** Quantification of p-srGAP2/input (n = 5 independent experiments. One-way analysis of variance was used for single-factor sample comparisons. ***P <​ 0.001). **(E)** Normalized CFP/FRET ratio of MDA-MB-231 cells individually expressing *srGAP2-M-cpstFRET*, *srGAP2 S80A-M-cpstFRET*, *srGAP2 S110A-M-cpstFRET*, *srGAP2 S206A-M-cpstFRET*or*srGAP2 S224A-M-cpstFRET* probe. One-way analysis of variance was used for single-factor sample comparisons. ***P <​ 0.001.** (F)** FRET representative images of MDA-MB-231 cells expressing *srGAP2-M-cpstFRET*, *srGAP2 S206A-M-cpstFRET* or *srGAP2 S206E-M-cpstFRET* probe. The number of filopodia per cell was quantified (length > 1 μm, n > 20 cells. One-way analysis of variance was used for single-factor sample comparisons. *P<​ 0.05 and ***P <​ 0.001).** (G)** FRET analysis of MDA-MB-231 cells expressing various* srGAP2* FRET probes in stiff-directed matrix. **(H)** Live cell imaging showed the migration tracks of randomly-selected cells in stiff-directed matrix within 1 h (n = 20 cells, 3 repeats).

As previous reports, the F-bar domain (membrane-binding domain) is mechanosensitive and could be under phosphorylated regulation 19. SrGAP2 was phosphorylated on serine residue in TNBC tissues, which was hardly observed in para-cancer tissues (Figure 5A). The phosphorylated form of srGAP2 was also found in the TNBC cell lines of MDA-MB-468 and MDA-MB-231, but not in the 293T and MCF7 cell lines (Figure 5B), suggesting srGAP2 phosphorylated modification closely associated with its tension role. Bioinformatics prediction showed that the sites most likely to be phosphorylated in the F-Bar domain were Ser^80^, Ser^110^, Ser^206^ and Ser^224^ (Figure S4A). Four srGAP2 mutated plasmids (srGAP2-S80A, srGAP2-S110A, srGAP2-S206A and srGAP2-S224A) were constructed to investigate the phosphorylation site of srGAP2. The results indicated that srGAP2-S206A cells dramatically down-regulated the phosphorylation levels of srGAP2 (p-srGAP2), while the three others maintained the same levels of p-srGAP2 as the wild-type group (Figure 5C-D).

Next, we investigated whether Ser^206^ mutation was involved in transmission of srGAP2 tension. We mutated the relevant Ser sites, S206A (de-phosphorylation of srGAP2 at Ser^206^) and S206E (pseudo-phosphorylation of srGAP2 at Ser^206^), of srGAP2 in the srGAP2-M-cpstFRET probe and confirmed these mutations not change srGAP2 binding to the cell membrane (Figure S2D). FRET analysis revealed that srGAP2 phosphorylation levels only at Ser^206^ were closely related to srGAP2 tension, essential for pseudopod formation (Figure 5E-F). Interestingly, srGAP2 de-phosphorylation (S206A) presented the dramatic tension decline, compared with the pseudo-phosphorylation (S206E) appeared strong srGAP2 tension (Figure 5F). For fast-moving cells in stiff-directed matrix, both de-phosphorylation and pseudo-phosphorylation mutations eliminated srGAP2 tension gradient (Figure 5G and Figure S5A). Cells carrying the srGAP2-S206A tension probe presented the loss of tension and disabled movement; while cells with the srGAP2-S206E tension probe showed even distribution of increased tension and abnormal direction recognition, although their movement speed was close to that of the wild type (Figure 5H). Based on the above results, the pulling effect of srGAP2 tension on the cell membrane is directly involved in membrane deformation, and the phosphorylation-induced srGAP2 tension gradient is the key to maintain cell directed migration to stiffer matrix.

### SrGAP2 Ser^206^ mutation suppresses TNBC cell invasion and metastasis *in vivo*

**Figure 6:**
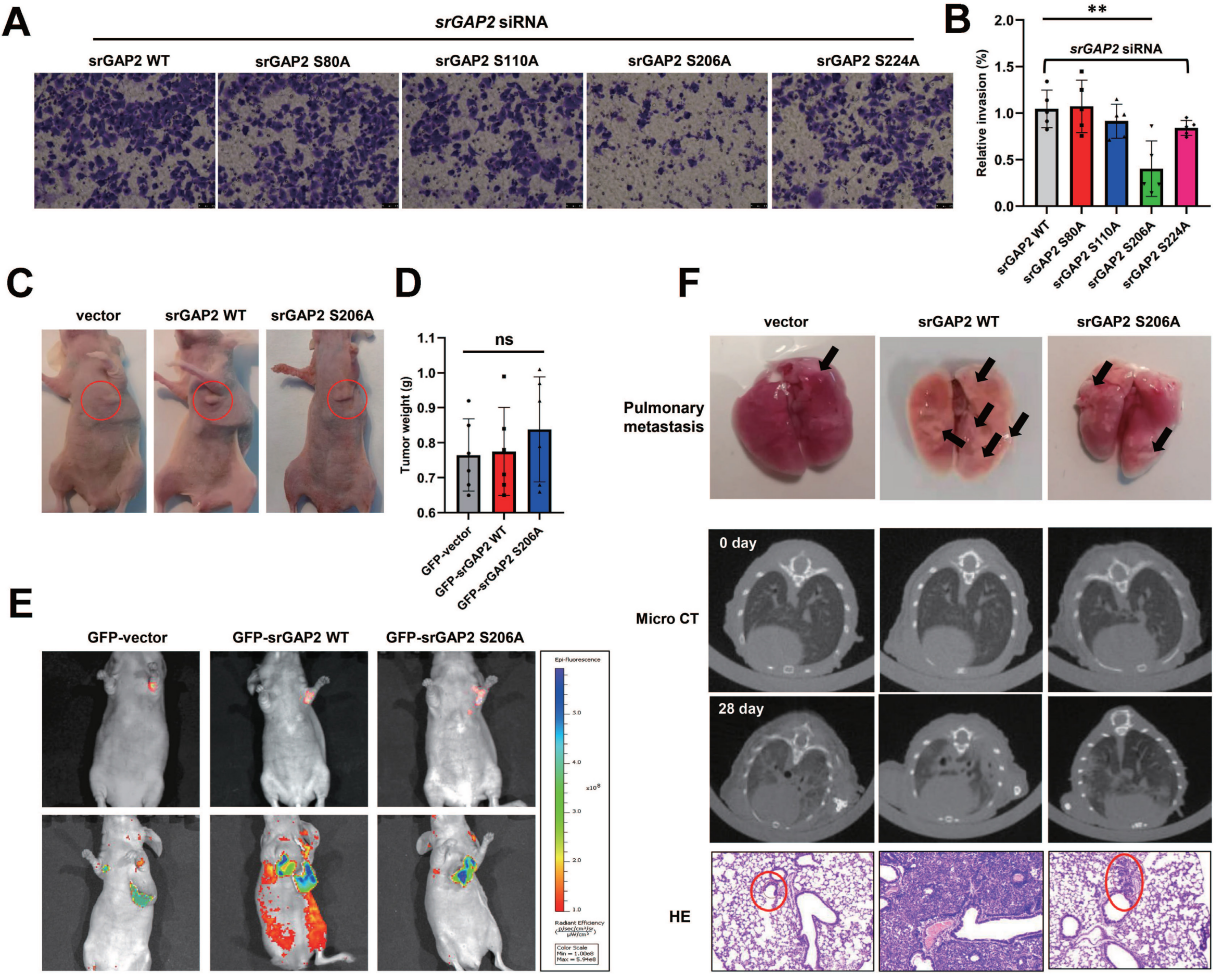
** SrGAP2 Ser206 phosphorylation involved in TNBC invasion *in vitro and in vivo.* (A)** and **(B)** *SrGAP2* was knocked down in TNBC cells that then transfected with *srGAP2* mutated plasmids. Invasion of TNBC cells was determined by the transwell invasion assay (mean ± SD, n = 5 experiments. One-way analysis of variance was used for single-factor sample comparisons. **P <​ 0.01). **(C)** Animals were randomly divided into three groups. Each group was injected *in situ* with the MDA-MB-231 cells stably expressing *GFP*-vector, *GFP-srGAP2 WT* and *GFP-srGAP2 S206A* (1 × 10^6^ cells suspended in 20 µL matrigel). On the 28th day, the mice were sacrificed. **(D)** Tumor weight. One-way analysis of variance was used for single-factor sample comparisons. **P <​ 0.01. ns: no statistical significance.** (E)** Bioluminescent images of systemic metastases in nude mice. **(F)** For the Orthotopic implantation assay, representative images of the typical lung tissues, the lung CT images, and H&E-stained histological sections of lungs were presented. The black arrow pointed to lung metastases in fresh lung tissue. The red circles marked metastatic nodules. One-way analysis of variance was used for single-factor sample comparisons. **P <​ 0.01. ns, no statistical significance.

To further clarify the effect of p-Ser206 dependent srGAP2 tension on TNBC cell aggressiveness, we transfected mutant srGAP2 plasmids (srGAP2-S80A, srGAP2-S110A, srGAP2-S206A and srGAP2-S224A) into srGAP2 knock-down MDA-MB-231 cells. Transwell assay showed that only srGAP2-S206A MDA-MB-231 cells displayed attenuated aggressiveness, while cells harboring the other three srGAP2 mutant plasmids were very aggressive, similar to srGAP2-WT cells (Figure 6A-B).

We next investigate the role of srGAP2 phosphorylation in TNBC aggressiveness *in vivo*, using GFP-tagged vector, srGAP2 WT and srGAP2 S206A cells. Orthotopic implantation assay was then performed by injecting the cells into mammary glands; there was no difference in tumor mass sizes among the three groups (Figure 6C-D). The mice in the srGAP2 WT group exhibited much more spontaneous metastatic signals than those in the vector control and srGAP2 S206A group (Figure 6E, ***P < 0.001). The micro-CT assay indicated that srGAP2-WT showed a marked pulmonary shadow and the number of intrapulmonary nodules in the srGAP2 WT group was significantly higher than that in the vector control and srGAP2 S206A group in lungs, as demonstrated by lung anatomy and H&E staining (Figure 6F). These results indicated that srGAP2 S206A phosphorylation is pivotal to promote the invasion and metastasis of TNBC cells *in vivo*.

### PKCα-mediated phosphorylation is responsible for matrix-directed polarization of srGAP2 tension

**Figure 7:**
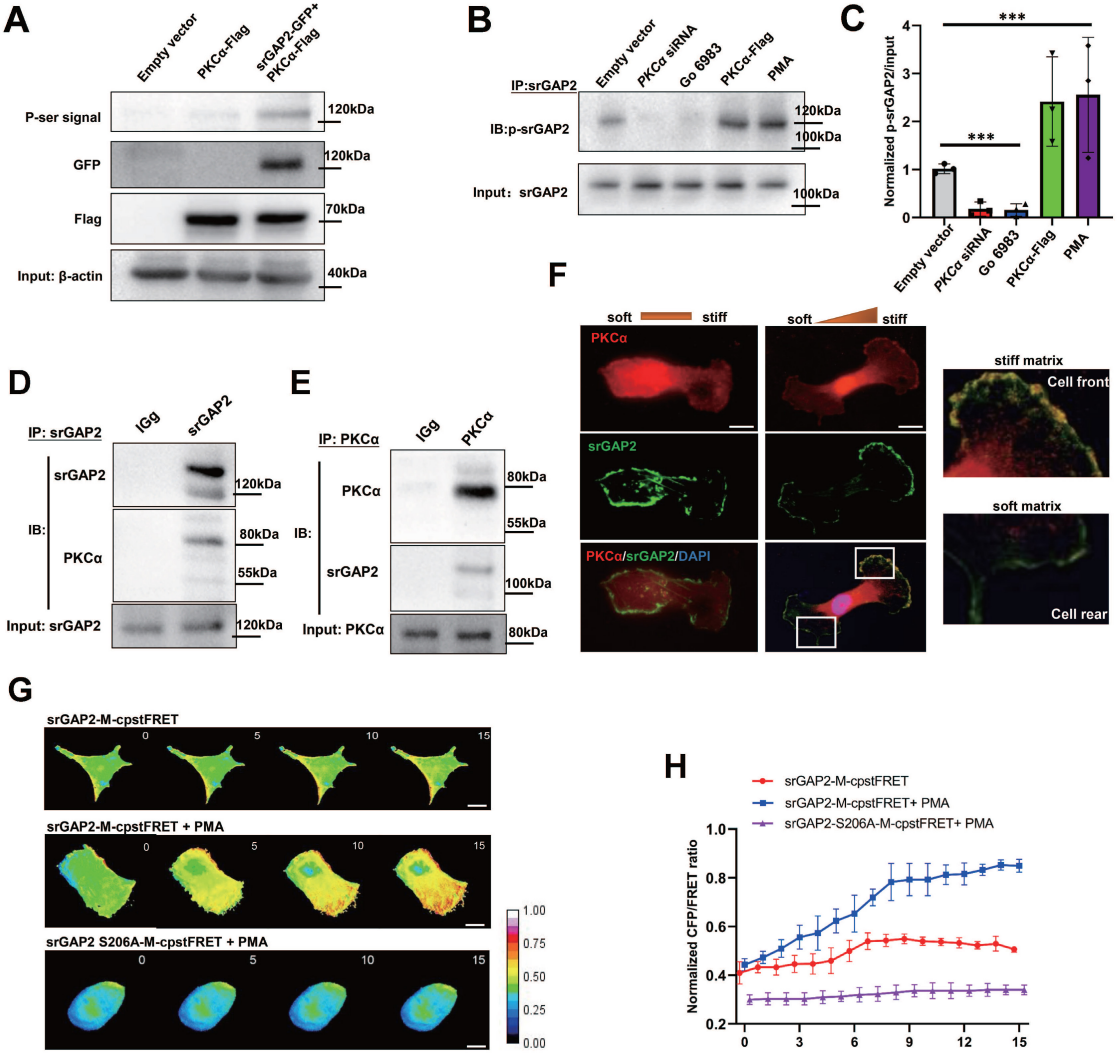
** SrGAP2 is phosphorylated by PKCα in invasive TNBC cells. (A)** Exogenous *srGAP2-GFP* and *PKCα-Flag* were expressed in 293T cells. The phospho-serine signals were detected with antibodies against phosphoserine. **(B)** The protein levels of PKCα in TNBC cells were determined by transfection with *PCKα* siRNA or *PKCα-Flag* plasmid. PKCα inhibitor (Go 6983, 10 μM) and PKCα agonists (PMA, 162 nM) was pre-incubated 30 min before the experiment. **(C)** Quantification of p-srGAP2/Input. One-way analysis of variance was used for single-factor sample comparisons. ***P <​ 0.001. **(D)** and **(E)** Endogenous PKCα interacted with srGAP2 in the fresh human TNBC tissues. **(F)** Cells moving across the matrix with or without stiff gradients. Immunofluorescence of endogenous PKCα and srGAP2 (White box: srGAP2 and PKCα distribution at front and rear of cells). **(G)** FRET time-lapse images of MDA-MB-231 cells expressing *srGAP2-M-cpstFRET* and *srGAP2ΔSH3-M-cpstFRET* probes treated with PMA for 15 min. **(H)** Normalized signals corresponding to srGAP2 tension and srGAP2 *ΔSH3* tension versus time, respectively (mean ± SEM, n ≥ 5 experiments).

It has been reported that other BDPs are phosphorylated by PKCα 36, next we investigated whether PKCα mediates srGAP2 phosphorylation, further regulating srGAP2 tension. In 293T cells without endogenous srGAP2, exogenously expressed PKCα strongly phosphorylated GFP-srGAP2 (Figure 7A). In MDA-MB-231 cells, endogenous srGAP2 phosphorylation was inhibited by *PKCα* siRNA or its inhibitor Go 6983; while srGAP2 phosphorylation was increased by PKCα overexpression or agonists PMA (Figure 7B-C). In human breast cancer tissue samples, co-IP assay showed strong binding of srGAP2 and PKCα (Figure 7D-E). More importantly, srGAP2 and PKCα colocalized in the forward part of matrix-directed moving cells, which was not observed when the matrix hardness was uniform, suggesting the polarized pattern of srGAP2 phosphorylation (Figure 7F). PMA, the agonist of PKCα, could enhance the srGAP2 tension with phosphorylated site, while de-phosphorylation mutated srGAP2 was out of PKCα and failed to tension activity (Figure 7G-H). These suggested PKCα-mediated srGAP2 phosphorylation displayed the cell-drived polarization, essential for strengthening srGAP2 tension and facilitating matrix-directed cell migration.

### PKCα is recruited by SDC4 at the cell front in stiff-directed matrix

**Figure 8:**
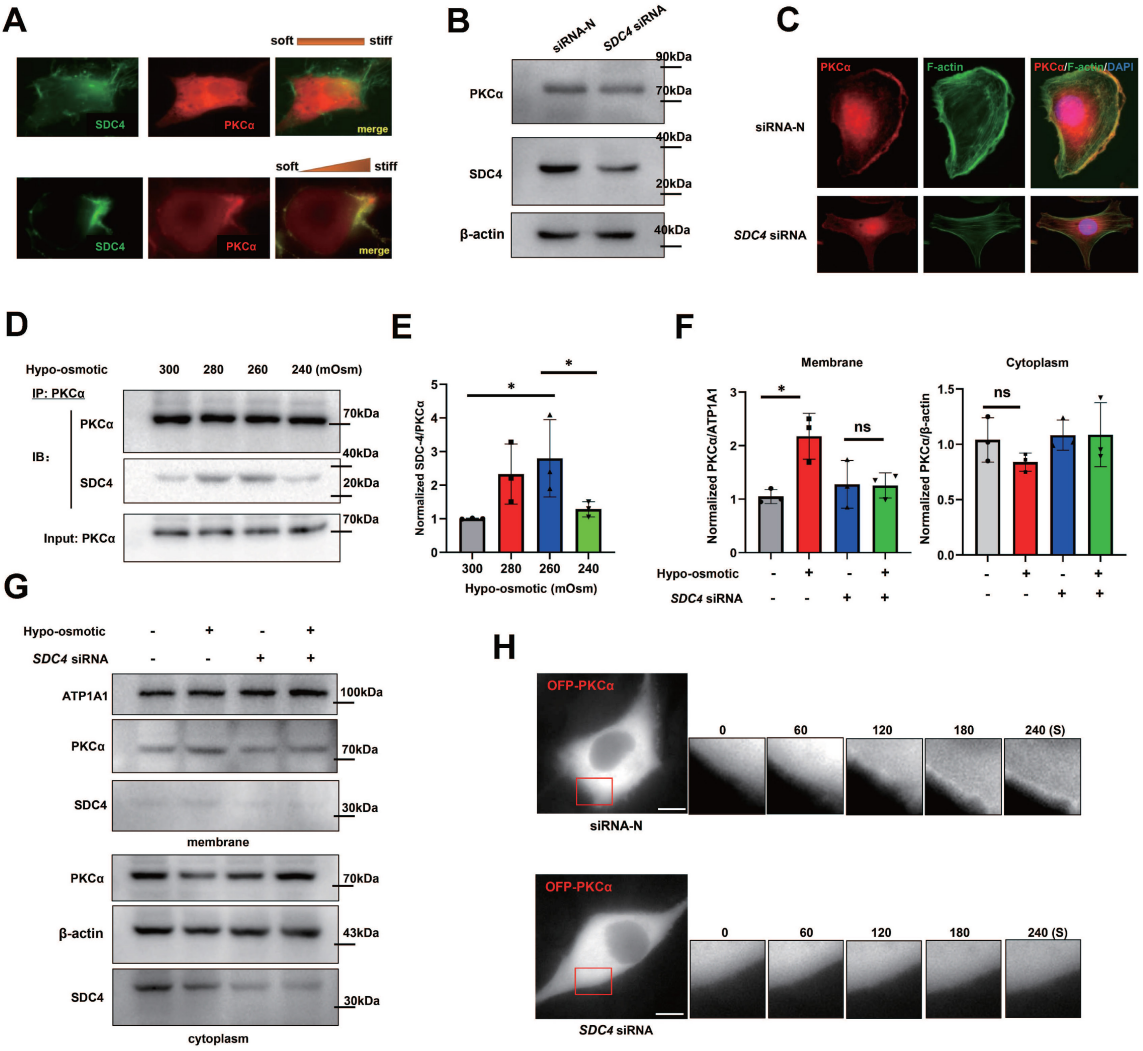
** PKCα is recruited by SDC4 at the cell front. (A)** TNBC cells transfected with *GFP-SDC4* and *OFP-PKCα* moving on the matrix with or without stiff gradients. **(B)** Immunoblot of *SDC4*-siRNA MDA-MB-231 cells. **(C)** Immunofluorescence of endogenous PKCα and F-actin. Nucleus was labeled with DAPI. **(D)** and** (E)** TNBC cells were treated with hypotonic or isotonic medium for 300 s and endogenous PKCα was immunoprecipitated. SDC4 and PKCα were identified by western blot (mean ± SD, n=3. One-way analysis of variance was used for single-factor sample comparisons. *P <​ 0.05).** (F)** and** (G)** Western blot analyses of PKCα membrane translocation in the hypo-osmotic-treated TNBC cells transfected with non-targeting siRNA or *SDC4* siRNA (mean ± SD, n= 3. hypo-osmotic: 260 mOsm. One-way analysis of variance was used for single-factor sample comparisons. *P <​ 0.05, ns: no statistical significance). ATP1A1 as membrane protein loading control, β-actin as cytoplasm protein loading control.** (H)** Time-lapse images of MDA-MB-231 cells co-transfected O*FP-PKCα* and on-targeting siRNA or *SDC4* siRNA in the hypotonic buffer for 300 s. Scale bar: 10 μm.

The transmembrane adhesive protein SDC4 can sense the ECM environment, and also activate the PKCα signaling pathway. In fast-moving cells, we found that SDC4 and PKCα co-located at the cell front (Figure 8A). To investigate whether SDC4 is involved in the PKCα polarized distribution in stiff-directed matrix, we down-regulated SDC4 expression in TNBC cells. However, SDC4 down-regulation does not seem to affect the PKCα protein levels (Figure 8B). In *SDC4*-siRNA TNBC cells, immunofluorescence assay showed that PKCα membrane translocation was significantly reduced under the stiff-matrix stimulation (Figure 8C).

Next, we doubt whether SDC4 could enhance PKCα recruitment under other mechanical stimuli. TNBC cells were treated with different hypotonic solutions of different concentrations, and co-immunoprecipitation results showed that the 260~280 mOsm hypotonic medium stimulation increased the binding of PKCα and SDC4, which disappeared when the osmotic pressure was too low (240 mOsm) (Figure 8D-E). In addition, PKCα membrane translocation was significantly increased in TNBC cells under the hypotonic stimulation, while this phenomenon was not observed in *SDC4*-siRNA cells (Figure 8F-G). Similar immunofluorescent results were found in cells transfected with OFP-PKCα plasmid (Figure 8H). All these suggested that the distinct extent of extracellular mechanics contributed to the regional recruitment of PKCα induced by SDC4.

### SDC4 is responsible for the membrane recruitment of PKCα under mechanical cues

**Figure 9:**
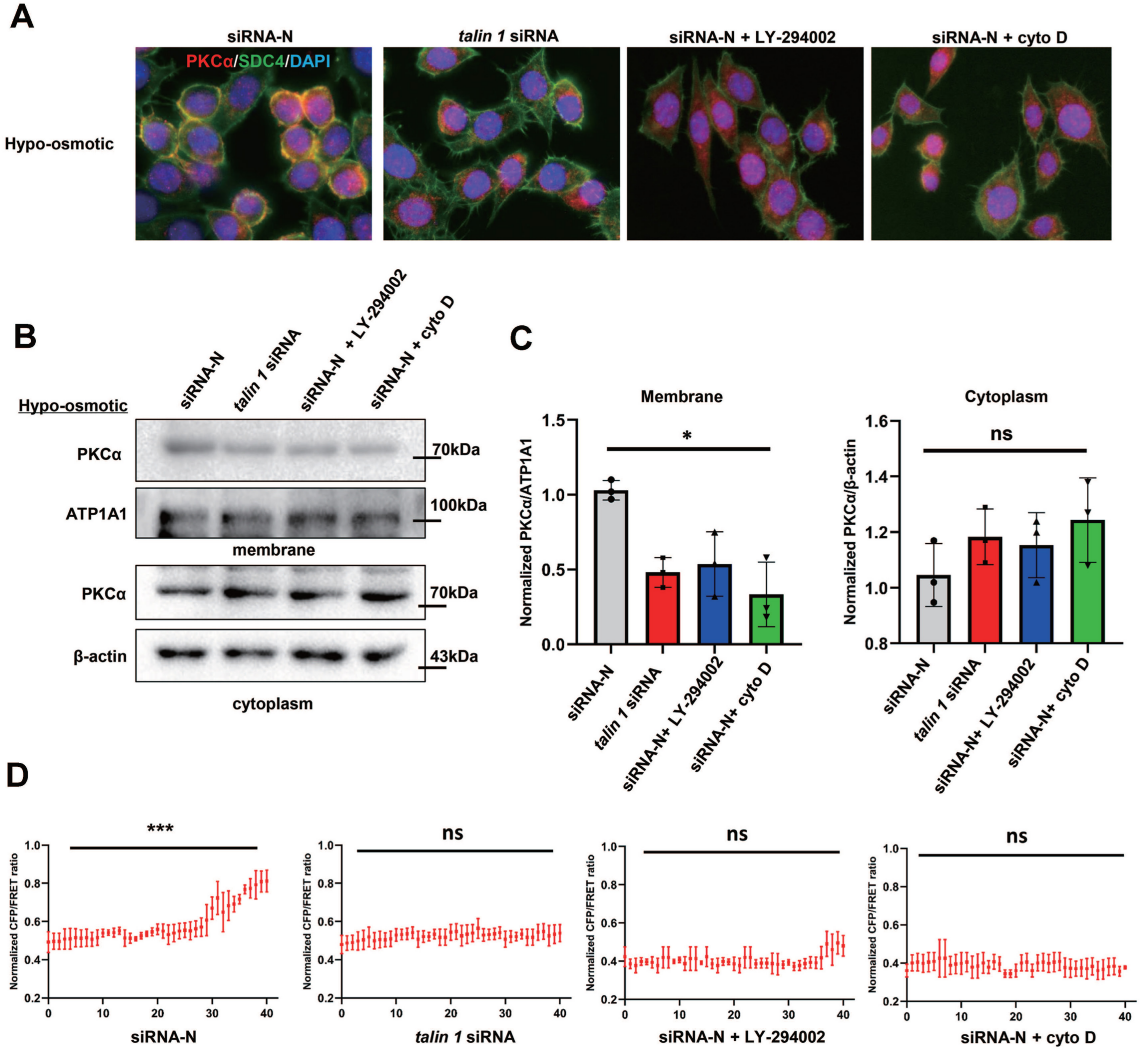
** Mechanical cues promoted PKCα recruitment by SDC4. (A)** Immunofluorescence of endogenous PKCα and SDC4 in TNBC cells treated with the hypotonic buffer for 300 s. Cells were transfected with on-targeting siRNA or *talin 1* siRNA. PI3K inhibitor (LY-294002, 30 μM) or Cytochalasin D (2 µM) was pre-incubated 30 min before the experiment. **(B)** and** (C)** Membrane translocation of PKCα in hypo-osmotic-treated TNBC cells, tested by western blot. ATP1A1 as membrane protein loading control, β-actin as cytoplasm protein loading control. Quantification of PKCα/ATP1A1 was showed (mean ± SD, n = 3. One-way analysis of variance was used for single-factor sample comparisons. *P <​ 0.05, ns: no statistical significance). ATP1A1 as membrane protein loading control, β-actin as cytoplasm protein loading control. **(D)** MDA-MB-231 cells expressing *srGAP2-M-cpstFRET* probes in stiff-directed matrix. CFP/FERT of the forty parts represented srGAP2 tension along with the moving direction.

It has been reported that the intracellular structure of SDC4 forms a protein complex with talin 1 that binds to F-actin. To investigate whether SDC4 tension is involved in PKCα recruitment, *TALIN 1* was knocked down in TNBC cells to weaken the connection between SDC4 and F-actin (Figure S2C, 18). Immunofluorescence assay showed that talin 1 down-regulation reduced the binding of SDC4 and PKCα on the cell membrane under the hypotonic stimulation. PI3K inhibitor LY-294002, which has been reported to reduce SDC4 tension, also decreased PKCα recruitment by SDC4. In addition, cyto D was used to depolymerize MF, and PKCα was unable to translocate to the membrane (Figure 9A). Similar results were observed in the PKCα protein levels on the membrane (Figure 9B-C). More importantly, when SDC tension was blocked, the increase of srGAP2 tension at the cell front in the stiff-directed matrix also disappeared (Figure 9D and Figure S5B).

### SDC4-PKCα signaling pathway phosphorylates srGAP2 *in vivo*

To further verify whether the SDC4-PKCα pathway regulates srGAP2 phosphorylation and tension *in vivo*, we investigated SDC4 binding to PKCα in the fresh human breast cancer tissues and adjacent tissues. The results showed that srGAP2 phosphorylation and SDC4-mediated PKCα recruitment were more obvious in cancer tissues compared with para tissues (Figure 5A and Figure S6A-B).

**Figure 10:**
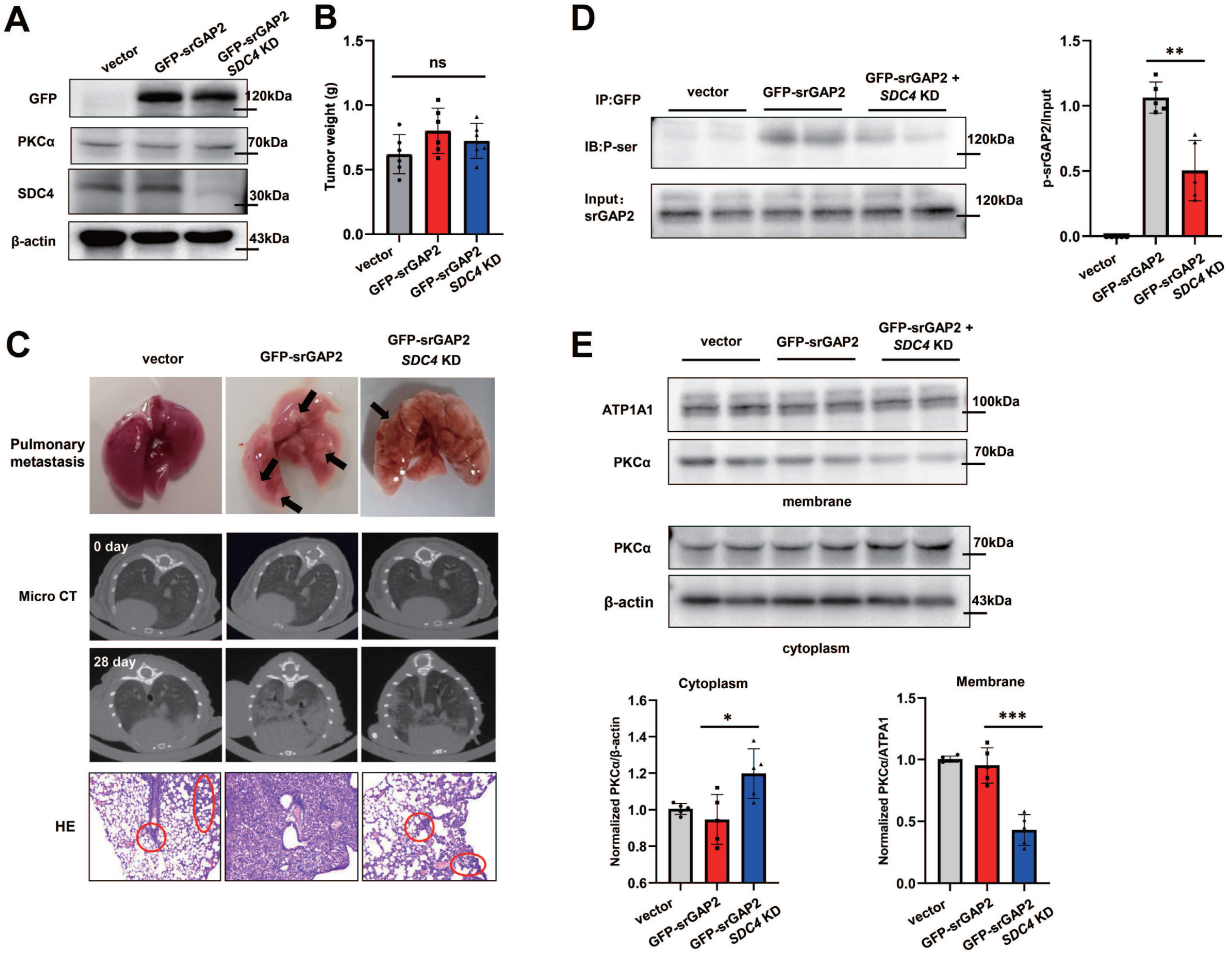
** SDC4-PKCα controls srGAP2 phosphorylation *in vivo.* (A)** Western blot of endogenous SDC4, PKCα and exogenous GFP-srGAP2 expression. β-actin as protein loading control. **(B)** Tumor weight. **(C)** For the Orthotopic implantation assay, representative images of the typical lung tissues, the lung CT images, and H&E-stained histological sections of lungs were presented. The black arrow pointed to lung metastases in the fresh lung tissues. The red circles marked metastatic nodules. **(D)** Endogenous srGAP2 was immunoprecipitated in the mice tumor tissues. Phosphoserine (p-Ser) and total srGAP2 were identified by western blot. Quantification of p-srGAP2/Input was showed (n = 5). **(E)** Western blot analysis of PKCα membrane translocation in the mice's tumor tissues. ATP1A1 as membrane protein loading control, β-actin as cytoplasm protein loading control. Quantification of PKCα/ATP1A1 was showed (n = 5). One-way analysis of variance was used for single-factor sample comparisons. *P <​ 0.05, **P <​ 0.01 and ***P <​ 0.001. ns, no statistical significance.

Next, we constructed MDA-MB-231 cell lines stably expressing different genotypes, including GFP-vector + NC; GFP-srGAP2 + NC; GFP-srGAP2 + *SDC4* KD (knock-down), and immunoblot assay showed that PKCα expression was not affected among the three groups (Figure 10A). The cells were then injected into nude mice mammary glands. After 28 days, there was no significant difference in tumor size among the three groups (Figure 10B). Analysis of lung metastasis of breast cancer in mice indicated that compared with GFP-srGAP2 + NC group, the lung metastasis was significantly reduced in GFP-srGAP2 + *SDC4* KD group (Figure 10C). Western Blot analysis of tumor tissue from the mice presented that, in GFP-srGAP2 + *SDC4* KD group, srGAP2 phosphorylation level and PKCα membrane translocation were significantly decreased (Figure 10D-E). These results suggested that srGAP2 phosphorylation was also controlled by SDC4-PKCα *in vivo*. In conclusion, we propose the mutually mechanical and chemical signals, including extracellular mechanical cues-sensory SDC4, the recruitment of PKCα on the regional membrane and phosphorylation-induced srGAP2 tension polarization, synergistically regulating the stiff matrix-directed cell migration.

## Discussion

Cells moving in the matrix can strain stiffen the micro-environment and self-adjust the stiff-directed migration. Extensive cell membrane deformation caused by intracellular mechanical forces is the key to fast move in the stiff matrix 37, 38. The present study has revealed how the cytoskeleton-membrane linker protein plays a tension role in regulating pseudopod formation and cell movement towards the stiff matrix mimic the tumor micro-environment. Notably, we have real-time monitored the polarized tension of mechanotransduction protein srGAP2 in living cells, which is implicated in the extra- and intracellular cascade of chemical and mechanical regulation, sensing ECM stiffening and promoting the matrix-directed migration.

Mechanical polarity is critical for stiff-directed cell migration. Multiple cytoskeleton related proteins present polarized recruits within moving cells. For example, nonmuscle myosin II isoforms organize cell front-rear polarity and promote rear contraction 39, 40; actin-related protein 2/3 mediated actin polymerization at the leading edge is required for migration of breast cancer cells in 3D matrix 41, 42. These studies suggest different mechanical properties potentially appear at the cell front and rear. The present study shows that srGAP2, as a force-transmitting linker, presents a mechanical polarity in fast-moving cells. Interestingly, the mechanical polarization did not depend on the srGAP2 protein translocation, but on the regional phosphorylated-modification of srGAP2. This phosphorylated srGAP2 with high mechanotransmitting capacity is expressed abundantly in the highly aggressive TNBC (Figure 5). Therefore, srGAP2-mediated mechanical polarization is involved in rapid migration of TNBC cells, particularly in the stiff-directed matrix.

Polarized srGAP2-induced cell migration could be due to control membrane remodeling and deformation. Membrane deformation requires the application of external mechanical forces derived from the abundance of actin binding proteins 43, 44, such as BDPs 9, act on the specific sites of cell membrane to overcome the high energy barrier of membrane deformation. Recent studies have reported shown that the cell front membrane softening is beneficial for tumor cells to go through the stiff interstitial matrix 45-47. In this study, srGAP2 tension increases only at the cell front, whose inward forces may be used to overcome the membrane barrier force and soften the membrane (Figure 4D). It has also been reported other cellular behaviors that force-transmitting linker proteins control membrane deformation. In yeast cells, endocytic adaptor sla2-mediated cytoskeletal tension acts directly on the membranes, resulting in membrane depression 48. In the process of cell proliferation, spectrin is required to attach cortical F-actin to cell membrane and support the appropriate cell morphology 32, 49.

We speculated that inward srGAP2 tension, coming from MF, promotes the outward expansion forces on filopodia formation. A reasonable theory would be that inward srGAP2 tension and outward expansion forces upregulate pressure density through their vector effect 50, 51. srGAP2 tension on the cell membrane was not uniformly enhanced upon stimulation with invasion-induced factors (EGF and CXCL12, Figure 4B-C), which is much higher at the filopodia. The mechanical heterogeneity of srGAP2 on the cell membrane implies that there exist pressure differences during filopodia formation. Cell membrane deformation requires inward forces, which can reduce the area of the front part of the protrusion, resulting in less resistance. Collectively, we speculate that srGAP2 acts as a regulator of outward expansion forces and is an essential force factor for pressure intensity.

Under the process of migration and invasion, cells are breaking through, and balancing the extra- and intracellular mechanical cues 52, 53. SDC4, a transmembrane heparan sulfate proteoglycan enriched in focal adhesion, could be mechanical sensor of micro-environment. It constructs link between extracellular matrix and intracellular signaling proteins 54. In stiff-directional cell migration, our study found that both SDC4 and PKCα are only clustered in the cell front, and knockdown of SDC4 expression could counteract the polarization of PKCα (Figure 8A and 8C). Therefore, SDC4 is an important link between the extracellular environment and the intracellular chemical-mechanical signaling pathway composed of PKCα and srGAP2.

Despite reports pointing out that, SDC4 particularly activates PKCα to bind on Phosphatidylinositol 4,5-bisphosphate (PIP2) at the V-region, forming a ternary complex 55. However, as a transmembrane tension-sensing protein, whether the mechanical tension of SDC4 is involved in PKCα activation remains to be clarified. We observe that appropriate mechanical stimulation (hypo-osmotic: 280~260 mOsm) promoted SDC4 recruitment of PKCα, while SDC4 lost the ability to recruit PKC when the mechanical force is too large. When SDC4 tension is attenuated, the binding ability of SDC4 to PKCα was also significantly reduced (Figure 9). These results suggest that local mechanical stimulation is the key to SDC4 recruitment of PKCα.

In summary, extra-intracellular mechanotransduction suffers under delicate regulation in the development of malignant invasion and metastasis. To break through the stiff matrix, srGAP2 exhibits the tension gradient from the leading to rear cell edges, the polarized tension highly dependent on the regional distribution of srGAP2 phosphorylation. This linker protein as the essential conveyor, whose tension is activated by the extracellular mechanical cues-induced SDC4-PKCα chemical modification, transmitted to intracellular MF forces, and applied on the membrane deformation. This polarized tension response sustains in the dynamic equilibrium and drives the bulleting cell migration in the stiff-matrix microenvironment. Targeting the srGAP2 phosphorylation and its mechanical transduction can develop therapeutic strategies of inhibiting TNBC cells durotaxis and new perspectives of the cancer treatment.

## Supplementary Material

Supplementary figures.

## Abbreviations

FRET: förster resonance energy transfer
srGAP2: slit-roundabout (Robo) GTPase-activating protein 2
BDPs: bar-domian proteins
ECM: extracellular matrix
MF: microfilament
TNBC: triple-negative breast cancer
CDM: cell-derived matrix
EGF: epidermal growth factor
CXCL12: C-X-C motif chemokine 12
PMA: phorbol 12-myristate 13-acetate
SDC4: syndecan-4
cyto D: cytochalasin D

## References

[1] Boyd NF, Dite GS, Stone J, Gunasekara A, English DR, McCredie MR (2002). Heritability of mammographic density, a risk factor for breast cancer. N Engl J Med.

[2] Northey JJ, Barrett AS, Acerbi I, Hayward MK, Talamantes S, Dean IS (2020). Stiff stroma increases breast cancer risk by inducing the oncogene ZNF217. J Clin Invest.

[3] Wei SC, Fattet L, Tsai JH, Guo Y, Pai VH, Majeski HE (2015). Matrix stiffness drives epithelial-mesenchymal transition and tumour metastasis through a TWIST1-G3BP2 mechanotransduction pathway. Nat Cell Biol.

[4] Tsujita K, Satow R, Asada S, Nakamura Y, Arnes L, Sako K (2021). Homeostatic membrane tension constrains cancer cell dissemination by counteracting BAR protein assembly. Nat Commun.

[5] Caswell PT, Zech T (2018). Actin-Based Cell Protrusion in a 3D Matrix. Trends Cell Biol.

[6] Lacy MM, Ma R, Ravindra NG, Berro J (2018). Molecular mechanisms of force production in clathrin-mediated endocytosis. FEBS Lett.

[7] Abella M, Andruck L, Malengo G, Skruzny M (2021). Actin-generated force applied during endocytosis measured by Sla2-based FRET tension sensors. Dev Cell.

[8] Li C, Chen L, Wang Y, Wang T, Di D, Zhang H (2021). Protein Nanoparticle-Related Osmotic Pressure Modifies Nonselective Permeability of the Blood-Brain Barrier by Increasing Membrane Fluidity. Int J Nanomedicine.

[9] Kapus A, Janmey P (2013). Plasma membrane-cortical cytoskeleton interactions: a cell biology approach with biophysical considerations. Compr Physiol.

[10] Scita G, Confalonieri S, Lappalainen P, Suetsugu S (2008). IRSp53: crossing the road of membrane and actin dynamics in the formation of membrane protrusions. Trends Cell Biol.

[11] Jian X, Brown P, Schuck P, Gruschus JM, Balbo A, Hinshaw JE (2009). Autoinhibition of Arf GTPase-activating protein activity by the BAR domain in ASAP1. J Biol Chem.

[12] Guerrier S, Coutinho-Budd J, Sassa T, Gresset A, Jordan NV, Chen K (2009). The F-BAR domain of srGAP2 induces membrane protrusions required for neuronal migration and morphogenesis. Cell.

[13] Li Y, Qiao L, Bai Y, Xiao C, Wu J, Gao X (2021). Identification of SRGAP2 as a potential oncogene and a prognostic biomarker in hepatocellular carcinoma. Life Sci.

[14] Lucas B, Hardin J (2017). Mind the (sr)GAP - roles of Slit-Robo GAPs in neurons, brains and beyond. J Cell Sci.

[15] Kuhn S, Geyer M (2014). Formins as effector proteins of Rho GTPases. Small GTPases.

[16] Hytonen VP, Wehrle-Haller B (2016). Mechanosensing in cell-matrix adhesions - Converting tension into chemical signals. Exp Cell Res.

[17] Okina E, Manon-Jensen T, Whiteford JR, Couchman JR (2009). Syndecan proteoglycan contributions to cytoskeletal organization and contractility. Scand J Med Sci Sports.

[18] Onyeisi JOS, Lopes CC, Gotte M (2021). Syndecan-4 as a Pathogenesis Factor and Therapeutic Target in Cancer. Biomolecules.

[19] Echarri A, Pavon DM, Sanchez S, Garcia-Garcia M, Calvo E, Huerta-Lopez C (2019). An Abl-FBP17 mechanosensing system couples local plasma membrane curvature and stress fiber remodeling during mechanoadaptation. Nat Commun.

[20] Senju Y, Suetsugu S (2015). Possible regulation of caveolar endocytosis and flattening by phosphorylation of F-BAR domain protein PACSIN2/Syndapin II. Bioarchitecture.

[21] Zhang J, Wang Y, Zheng Z, Sun X, Chen T, Li C (2019). Intracellular ion and protein nanoparticle-induced osmotic pressure modify astrocyte swelling and brain edema in response to glutamate stimuli. Redox Biol.

[22] Hu Y, Xie Q, Chen S, Zhao W, Zhao X, Ruan Q (2022). Par3 promotes breast cancer invasion and migration through pull tension and protein nanoparticle-induced osmotic pressure. Biomed Pharmacother.

[23] Zhang X, Li G, Guo Y, Song Y, Chen L, Ruan Q (2019). Regulation of ezrin tension by S-nitrosylation mediates non-small cell lung cancer invasion and metastasis. Theranostics.

[24] Schindelin J, Arganda-Carreras I, Frise E, Kaynig V, Longair M, Pietzsch T (2012). Fiji: an open-source platform for biological-image analysis. Nat Methods.

[25] Hu Y, Xie Q, Wu X, Liu W, Li D, Li C (2022). Tension of plus-end tracking protein Clip170 confers directionality and aggressiveness during breast cancer migration. Cell Death Dis.

[26] Caswell PT, Spence HJ, Parsons M, White DP, Clark K, Cheng KW (2007). Rab25 associates with alpha5beta1 integrin to promote invasive migration in 3D microenvironments. Dev Cell.

[27] Cukierman E, Pankov R, Stevens DR, Yamada KM (2001). Taking cell-matrix adhesions to the third dimension. Science.

[28] Petrie RJ, Gavara N, Chadwick RS, Yamada KM (2012). Nonpolarized signaling reveals two distinct modes of 3D cell migration. J Cell Biol.

[29] Wang S, Matsumoto K, Lish SR, Cartagena-Rivera AX, Yamada KM (2021). Budding epithelial morphogenesis driven by cell-matrix versus cell-cell adhesion. Cell.

[30] Hetmanski JHR, de Belly H, Busnelli I, Waring T, Nair RV, Sokleva V (2019). Membrane Tension Orchestrates Rear Retraction in Matrix-Directed Cell Migration. Dev Cell.

[31] Doherty GJ, McMahon HT (2008). Mediation, modulation, and consequences of membrane-cytoskeleton interactions. Annu Rev Biophys.

[32] Deng H, Yang L, Wen P, Lei H, Blount P, Pan D (2020). Spectrin couples cell shape, cortical tension, and Hippo signaling in retinal epithelial morphogenesis. J Cell Biol.

[33] Naj X, Hoffmann AK, Himmel M, Linder S (2013). The formins FMNL1 and mDia1 regulate coiling phagocytosis of Borrelia burgdorferi by primary human macrophages. Infect Immun.

[34] Azios NG, Krishnamoorthy L, Harris M, Cubano LA, Cammer M, Dharmawardhane SF (2007). Estrogen and resveratrol regulate Rac and Cdc42 signaling to the actin cytoskeleton of metastatic breast cancer cells. Neoplasia.

[35] Mukherjee D, Zhao J (2013). The Role of chemokine receptor CXCR4 in breast cancer metastasis. Am J Cancer Res.

[36] Nguyen LP, Tran SC, Suetsugu S, Lim YS, Hwang SB (2020). PACSIN2 Interacts with Nonstructural Protein 5A and Regulates Hepatitis C Virus Assembly. J Virol.

[37] Torrino S, Grasset EM, Audebert S, Belhadj I, Lacoux C, Haynes M (2021). Mechano-induced cell metabolism promotes microtubule glutamylation to force metastasis. Cell Metab.

[38] van Helvert S, Friedl P (2016). Strain Stiffening of Fibrillar Collagen during Individual and Collective Cell Migration Identified by AFM Nanoindentation. ACS Appl Mater Interfaces.

[39] Vicente-Manzanares M, Ma X, Adelstein RS, Horwitz AR (2009). Non-muscle myosin II takes centre stage in cell adhesion and migration. Nat Rev Mol Cell Biol.

[40] Raab M, Swift J, Dingal PC, Shah P, Shin JW, Discher DE (2012). Crawling from soft to stiff matrix polarizes the cytoskeleton and phosphoregulates myosin-II heavy chain. J Cell Biol.

[41] Romero S, Le Clainche C, Gautreau AM (2020). Actin polymerization downstream of integrins: signaling pathways and mechanotransduction. Biochem J.

[42] Frugtniet B, Jiang WG, Martin TA (2015). Role of the WASP and WAVE family proteins in breast cancer invasion and metastasis. Breast Cancer (Dove Med Press).

[43] Johannes L, Wunder C, Bassereau P (2014). Bending "on the rocks"-a cocktail of biophysical modules to build endocytic pathways. Cold Spring Harb Perspect Biol.

[44] Stachowiak JC, Brodsky FM, Miller EA (2013). A cost-benefit analysis of the physical mechanisms of membrane curvature. Nat Cell Biol.

[45] Schierbaum N, Rheinlaender J, Schaffer TE (2017). Viscoelastic properties of normal and cancerous human breast cells are affected differently by contact to adjacent cells. Acta Biomater.

[46] Pontes B, Monzo P, Gauthier NC (2017). Membrane tension: A challenging but universal physical parameter in cell biology. Semin Cell Dev Biol.

[47] Tian M, Li Y, Liu W, Jin L, Jiang X, Wang X (2015). The nanomechanical signature of liver cancer tissues and its molecular origin. Nanoscale.

[48] Messa M, Fernandez-Busnadiego R, Sun EW, Chen H, Czapla H, Wrasman K (2014). Epsin deficiency impairs endocytosis by stalling the actin-dependent invagination of endocytic clathrin-coated pits. Elife.

[49] Machnicka B, Grochowalska R, Boguslawska DM, Sikorski AF, Lecomte MC (2012). Spectrin-based skeleton as an actor in cell signaling. Cell Mol Life Sci.

[50] Guo YC, Wang YX, Ge YP, Yu LJ, Guo J (2018). Analysis of subcellular structural tension in axonal growth of neurons. Rev Neurosci.

[51] Chen T, Guo Y, Shan J, Zhang J, Shen X, Guo J (2019). Vector Analysis of Cytoskeletal Structural Tension and the Mechanisms that Underpin Spectrin-Related Forces in Pyroptosis. Antioxid Redox Signal.

[52] Barriga EH, Franze K, Charras G, Mayor R (2018). Tissue stiffening coordinates morphogenesis by triggering collective cell migration *in vivo*. Nature.

[53] Discher DE, Janmey P, Wang YL (2005). Tissue cells feel and respond to the stiffness of their substrate. Science.

[54] Gopal S, Multhaupt HAB, Pocock R, Couchman JR (2017). Cell-extracellular matrix and cell-cell adhesion are linked by syndecan-4. Matrix Biol.

[55] Chronopoulos A, Thorpe SD, Cortes E, Lachowski D, Rice AJ, Mykuliak VV (2020). Syndecan-4 tunes cell mechanics by activating the kindlin-integrin-RhoA pathway. Nat Mater.

