# Supplementary material for: Stiff matrix induced srGAP2 tension gradients control migration direction in triple-negative breast cancer
Source: Theranostics. 2023 Jan 1;13(1):59–76. doi: 10.7150/thno.77313 (PMC9800732; doi:10.7150/thno.77313)
Supplement: Supplementary file 1 — Supplementary figures. [file thnov13p0059s1.pdf]

Supplementary materials

Figure Legends

Figure S1 *SRGAP2* expression is correlated with tumors malignant behavior

(A) *SRGAP2* expression levels among tumor tissues and normal tissues from the Cancer Genome Atlas database. **(B)** Kaplan-Meier plots of overall survival of patients with breast cancer and triple-negative breast cancer.

Figure S2 SrGAP2 protein expression did not affect MF polymerization

(A) Mean ratios of F-actin/G-actin in TNBC cells transfected with pCMV-N or pCMV-srGAP2. No statistical difference between the two groups. **(B)** Actin tension probes were expressed in TNBC cells of control group, srGAP2 knockdown group and srGAP2 overexpression group. No statistical difference in actin tension among the three groups. **(C)** Immunoblot of MDA-MB-231 cells knocked down for FMNL1 and FMNL3. **(D)** Immunoblot of MDA-MB-231 cells knocked down for talin 1. **(E)** TNBC cells expressed GFP-srGAP2 WT, GFP-srGAP2 S206A and GFP-srGAP2 S206E.

Figure S3 Conformation of srGAP2 tension probes and their effective analysis

(A) The probe model consists of srGAP2-M-cpstFRET. The cpVenus is parallel to the cpCerulean when an external force across cpstFRET generates a certain angle, reducing FRET efficiency. **(B)** Insertion position of the probes in srGAP2 structure. **(C)** Testing the reliability of the srGAP2-M-cpstFRET probe using the acceptor bleaching FRET and fluorescence recovery after photobleaching tests. **(D)** and **(E)** Representative image of MDA-MB-231 cells expressing srGAP2-M-cpstFRET that recovered over time after laser bleaching. red box: bleached area. Normalized average fluorescence recovery of srGAP2-M-cpstFRET versus time (n = 8). Scale bar, 20 μm.

Figure S4 (A) Bioinformatics predicted serine phosphorylation sites in the F-Bar domain of srGAP2. Data from https://services.healthtech.dtu.dk

Figure S5 Representative images of TNBC cell expressing srGAP2-M-cpstFRET probes

(A) Representative images of TNBC cell expressing srGAP2-M-cpstFRET, srGAP2 S206A-M-cpstFRET and srGAP2 S206E-M-cpstFRET. The calibration bar: 0.3 to 0.9. **(B)** Representative images of TNBC cell expressing srGAP2-M-cpstFRET transfected with N siRNA or talin1 siRNA. PI3K inhibitor (LY-294002, 30 μM) and Cytochalasin D (2 μM) was pre-incubated 30 mins before the experiment.

Figure S6 (A) and **(B)** Endogenous PKCα interacted with SDC4 in the fresh human TNBC tissues and para tissues.

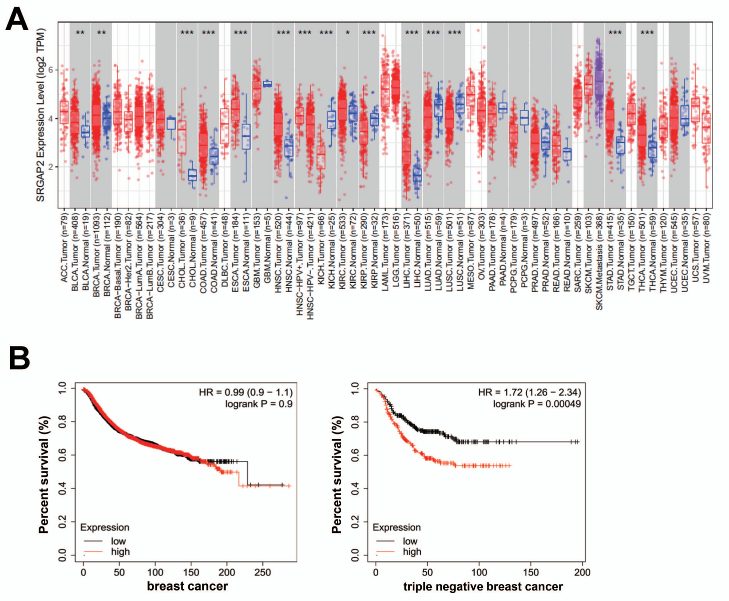
A
B
SRGAP2 Expression Level (log2 TPM)
** ** *** *** *** *** *** *** * *** *** *** *** *** ***
ACC.Tumor (n=79)
BLCA.Tumor (n=408)
BLCA.Normal (n=19)
BRCA.Tumor (n=1093)
BRCA.Normal (n=112)
BRCA−Basal.Tumor (n=190)
BRCA−Her2.Tumor (n=82)
BRCA−LumA.Tumor (n=564)
BRCA−LumB.Tumor (n=217)
CESC.Tumor (n=304)
CESC.Normal (n=3)
CHOL.Tumor (n=36)
CHOL.Normal (n=9)
COAD.Tumor (n=457)
COAD.Normal (n=41)
DLBC.Tumor (n=48)
ESCA.Tumor (n=184)
ESCA.Normal (n=11)
GBM.Tumor (n=153)
GBM.Normal (n=5)
HNSC.Tumor (n=520)
HNSC.Normal (n=44)
HNSC−HPV+.Tumor (n=97)
HNSC−HPV−.Tumor (n=421)
KICH.Tumor (n=66)
KICH.Normal (n=25)
KIRC.Tumor (n=533)
KIRC.Normal (n=72)
KIRP.Tumor (n=290)
KIRP.Normal (n=32)
LAML.Tumor (n=173)
LGG.Tumor (n=516)
LIHC.Tumor (n=371)
LIHC.Normal (n=50)
LUAD.Tumor (n=515)
LUAD.Normal (n=59)
LUSC.Tumor (n=501)
LUSC.Normal (n=51)
MESO.Tumor (n=87)
OV.Tumor (n=303)
PAAD.Tumor (n=178)
PAAD.Normal (n=4)
PCPG.Tumor (n=179)
PCPG.Normal (n=3)
PRAD.Tumor (n=497)
PRAD.Normal (n=52)
READ.Tumor (n=166)
READ.Normal (n=10)
SARC.Tumor (n=259)
SKCM.Tumor (n=103)
SKCM.Metastasis (n=368)
STAD.Tumor (n=415)
STAD.Normal (n=35)
TGCT.Tumor (n=150)
THCA.Tumor (n=501)
THCA.Normal (n=59)
THYM.Tumor (n=120)
UCEC.Tumor (n=545)
UCEC.Normal (n=35)
UCS.Tumor (n=57)
UVM.Tumor (n=80)
HR = 0.99 (0.9 − 1.1)
logrank P = 0.9
Percent survival (%)
Expression
low
high
breast cancer
HR = 1.72 (1.26 − 2.34)
logrank P = 0.00049
Percent survival (%)
Expression
low
high
triple negative breast cancer

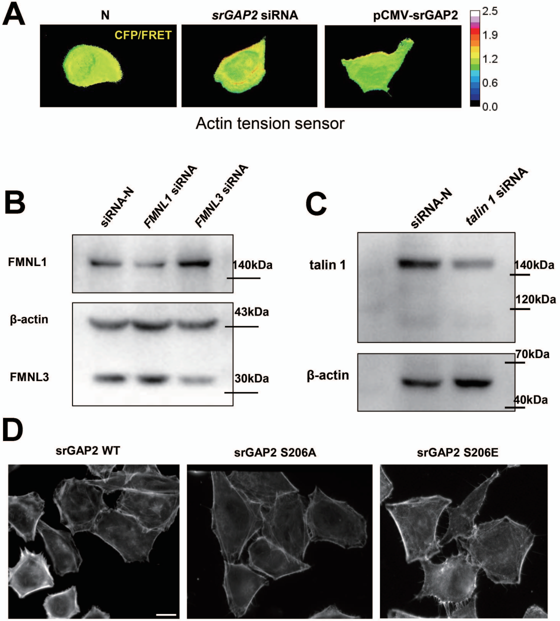
A
N
srGAP2 siRNA
pCMV-srGAP2
CFP/FRET
2.5
1.9
1.2
0.6
0.0
Actin tension sensor
B
siRNA-N
FMNL1 siRNA
FMNL3 siRNA
FMNL1
140kDa
β-actin
43kDa
FMNL3
30kDa
C
siRNA-N
talin 1 siRNA
talin 1
140kDa
120kDa
70kDa
β-actin
40kDa
D
srGAP2 WT
srGAP2 S206A
srGAP2 S206E

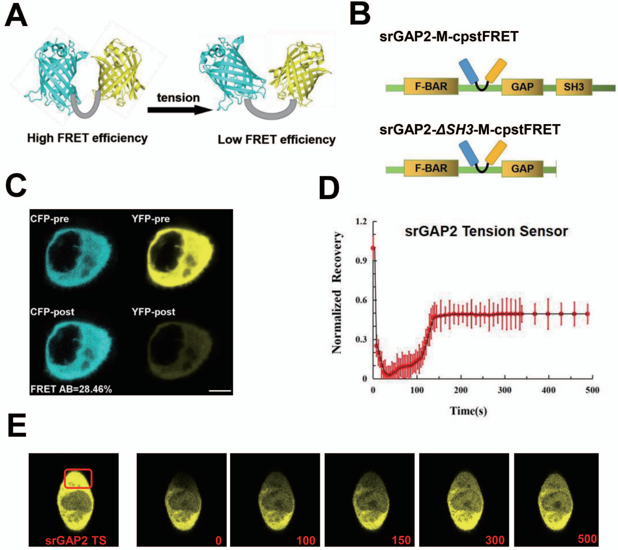

A
tension
High FRET efficiency
Low FRET efficiency
B
srGAP2-M-cpstFRET
F-BAR
GAP
SH3
srGAP2-ΔSH3-M-cpstFRET
F-BAR
GAP
C
CFP-pre
YFP-pre
CFP-post
YFP-post
FRET AB=28.46%
D
srGAP2 Tension Sensor
Normalized Recovery
Time(s)
0
100
200
300
400
500
0
0.3
0.6
0.9
1.2
E
srGAP2 TS
0
100
150
300
500

A

SITE	CONTEXT	SCORE
**64 S**	EMDYSRNLE	0.507
**80 S**	AKTRSTKDQ	0.996
**94 S**	QNVLSPVNC	0.547
**110 S**	VKRESRDHT	0.991
**117 S**	HTTLSDIYL	0.593
**132 S**	FVQVSEDSG	0.941
**135 S**	VSEDSGRLF	0.715
**142 S**	LFKKSKEVG	0.874
**162 S**	NELYSVMKT	0.453
**174 S**	YNADSISAQ	0.827
**176 S**	ADSISAQSK	0.443
**179 S**	ISAQSKLKE	0.601
**195 S**	EMDYSRNLE	0.507
**206 S**	AKTRSTKDQ	0.996
**209 S**	QNVLSPVNC	0.547
**224 S**	VKRESRDHT	0.991
**263 S**	HTTLSDIYL	0.593
**273 S**	FVQVSEDSG	0.941
**287 S**	VSEDSGRLF	0.715
**297 S**	LFKKSKEVG	0.874
**305 S**	NELYSVMKT	0.453

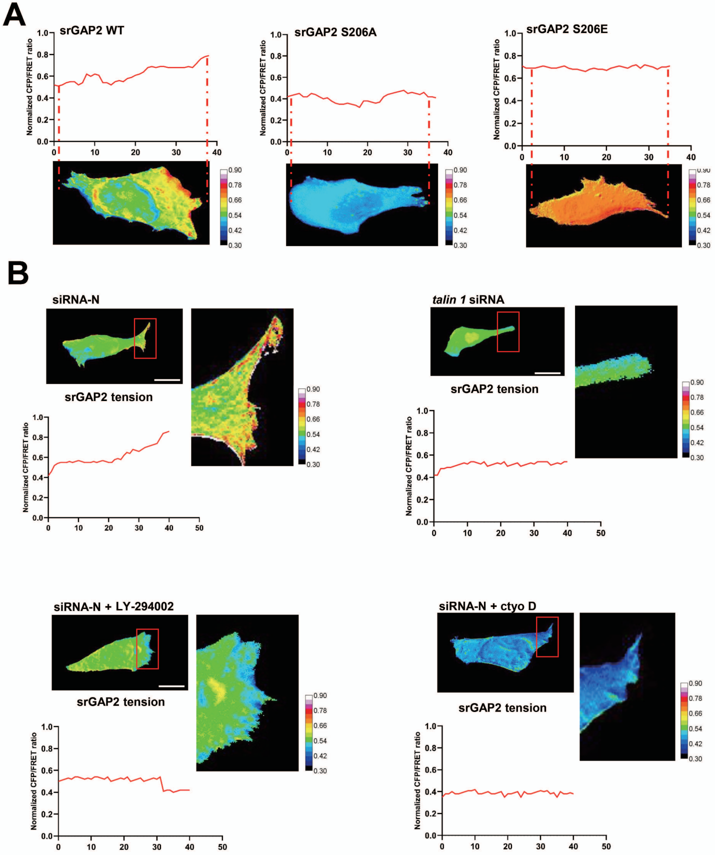
A
srGAP2 WT
srGAP2 S206A
srGAP2 S206E
Normalized CFP/FRET ratio
0.90
0.78
0.66
0.54
0.42
0.30
B
siRNA-N
srGAP2 tension
talin 1 siRNA
srGAP2 tension
siRNA-N + LY-294002
srGAP2 tension
siRNA-N + ctyo D
srGAP2 tension

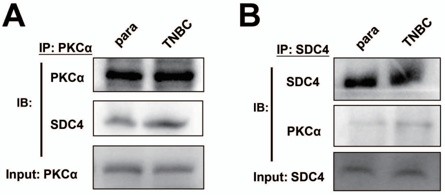
A
IP: PKCα
para
TNBC
IB:
PKCα
SDC4
Input: PKCα
B
IP: SDC4
para
TNBC
IB:
SDC4
PKCα
Input: SDC4
